# Breeding vegetables for whitefly resistance: past, present, and future in the AI era

**DOI:** 10.3389/fpls.2025.1724403

**Published:** 2026-01-30

**Authors:** Deepa Jaganathan, Bhabesh Dutta, Saumik Basu, Sudeep Bag, Rajagopalbabu Srinivasan, Assaf Eybishitz, Derek W. Barchenger, Lotte Caarls, Alvin M. Simmons, Amol N. Nankar

**Affiliations:** 1 Department of Horticulture, College of Agricultural and Environmental Sciences, University of Georgia, Tifton, GA, United States; 2 Institute of Plant Breeding, Genetics, and Genomics, College of Agricultural and Environmental Sciences (CAES), University of Georgia, Tifton, GA, United States; 3 Department of Plant Pathology, College of Agricultural and Environmental Sciences, University of Georgia, Tifton, GA, United States; 4 Department of Entomology, College of Agricultural and Environmental Sciences, University of Georgia, Tifton, GA, United States; 5 Department of Entomology, College of Agricultural and Environmental Sciences, University of Georgia, Griffin, GA, United States; 6 World Vegetable Center, Tainan, Taiwan; 7 Laboratory of Entomology, Plant Breeding Wageningen University & Research (WUR), Wageningen, Netherlands; 8 United States Vegetable Laboratory, Agricultural Research Service, United States Department of Agriculture (USDA), Charleston, SC, United States

**Keywords:** artificial intelligence in plant breeding, genomic selection, host resistance, marker-assisted selection (MAS), multiomics, vegetable breeding

## Abstract

Whiteflies, particularly *Bemisia tabaci*—a rapidly evolving cryptic species complex comprising more than 40 biotypes including the invasive MEAM1 and MED—and *Trialeurodes vaporariorum*, remain among the most destructive pests of global vegetable production. Their adaptability, wide host range, and efficient virus transmission drive recurrent epidemics in crops such as tomato, pepper, eggplant, cucurbits, and snapbean. Over six decades, breeding for whitefly resistance has progressed from phenotypic selection to the identification of resistance mechanisms such as antibiosis, antixenosis, and tolerance, and to the exploitation of diverse sources from wild relatives and landraces. Recent advances in QTL mapping, pangenomics, multi-omics integration, genomic selection, and CRISPR-based modification of metabolic and structural defense traits have transformed the landscape of resistance breeding. Emerging AI-enabled tools—including machine-learning models for automated whitefly phenotype detection, hyperspectral stress diagnostics, and predictive modelling of resistance loci—are accelerating the dissection and deployment of complex traits. Importantly, durable whitefly resistance enhances climate resilience by reducing dependence on insecticides, stabilizing yields under abiotic–biotic stress combinations, and mitigating climate-driven surges in whitefly populations and virus epidemics. By integrating classical genetics, modern biotechnology, multi-omics, and AI-driven decision frameworks, breeding programs can more rapidly develop robust, climate-resilient vegetable cultivars capable of withstanding evolving whitefly threats.

## Graphical Abstract

**Figure:**
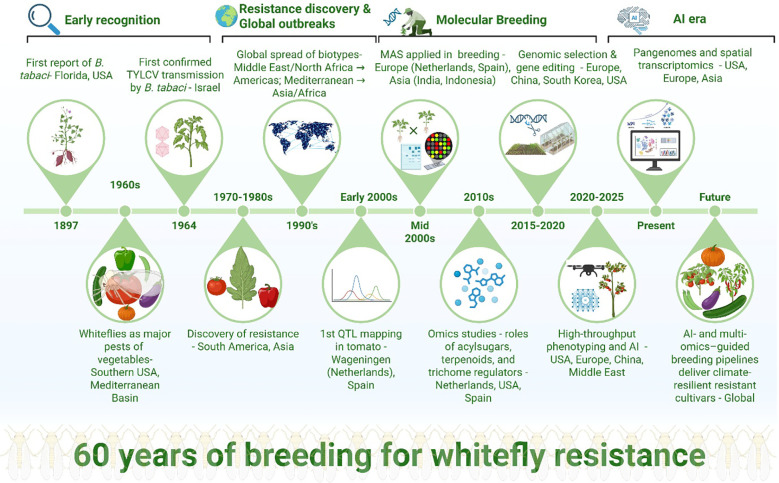

## Whitefly problems in global agriculture production

Whiteflies (Hemiptera: Aleyrodidae), particularly *Bemisia tabaci* G and *Trialeurodes vaporariorum*, are among the most devastating insect pests affecting global vegetable production, with significant economic consequences for crops such as cucurbits, tomato (*Solanum lycopersicum*), pepper (*Capsicum annuum*), eggplant (*S. melongena*), and snapbean (*Phaseolus vulgaris*). The global success of whiteflies can be attributed to a range of adaptive characteristics, including polyphagy, high fecundity, short generation time, and an exceptional ability to develop resistance to multiple classes of insecticides. Additionally, the presence of cryptic species within *B. tabaci* facilitates ecological specialization and enhances adaptability across diverse environments. The species complex of *B. tabaci* is by far the most problematic issue from whiteflies on a global scale. With a host range of over 1000 plant species for *B. tabaci*, the relative acceptance and performance vary among and within these plant species (Simmons et al., 2008, Abd-Rabou and Simmons, 2010; Olaniyi et al., 2021). Whitefly damage is twofold: direct feeding on phloem sap weakens plant vigor, induces chlorosis, leaf wilting, stunted growth and honeydew accumulation—leading to sooty mold. Indirect effects arise from their role as vectors of over 300 plant viruses (Navas-Castillo et al., 2011). These include the tomato yellow leaf curl virus (TYLCV), chili leaf curl virus (ChiLCV), pepper yellow leaf curl virus (PepYLCV), pepper yellow mosaic virus and bean golden mosaic virus (BGMV). The efficiency of virus spread is strongly influenced by transmission biology. Most begomoviruses, including TYLCV, are transmitted in a persistent–circulative manner: virions are ingested, circulate through the hemolymph, and accumulate in the salivary glands, enabling long-term infectivity for days to weeks (Ghosh and Ghanim, 2021). Other whitefly-transmitted viruses exhibit semi-persistent transmission, where virions adhere to the foregut without circulation, or non-persistent transmission, where spread occurs rapidly during brief exploratory probes (Naveed et al., 2023). These contrasting mechanisms underpin differences in epidemic velocity and the difficulty of controlling virus outbreaks in vegetable crops (Legarrea et al., 2015). The combined impact of whitefly infestation and virus transmission continues to challenge profitability and productivity in tomato-growing regions (Fonsah et al., 2018). Whiteflies, including *B. tabaci* and *T. vaporariorum*, were documented as significant pests of horticultural crops in the United States in the late 19th century (Quaintance, 1900). That was the first report in the United States of B. *tabaci* (reported as *Aleyrodes inconspicua*); it was found in the state of Florida feeding on sweetpotato (*Ipomoea batatas*) and *Physalis* sp. (a solanaceous weed) in 1897, and on cultivated okra (*Abelmoschus esculentus*) in 1898 (Quaintance, 1900). Their status as important pests of tomato and other vegetables was recognized during the mid-20^th^ century as intensive vegetable cultivation expanded globally (Brown et al., 1995; Byrne and Bellows, 1991). Since then, *B. tabaci* has emerged as a global pest, from their feeding virus transmission. Since the first description of TYLCV transmission by *B. tabaci* by Cohen and Harpaz (1964), these pests have intensified their threat, expanding their geographic range and ecological niches, aided by global seed trade, intensive cultivation, and warming climates. Historically, whitefly management in vegetable crops heavily relied upon chemical insecticides (Horowitz et al., 2020). In addition to chemical and genetic approaches, integrated pest management (IPM) strategies increasingly rely on biological control agents such as *Encarsia formosa*, *Eretmocerus eremicus*, predatory mites, and lacewings, which suppress whitefly populations through parasitism and predation (van Lenteren et al., 1995; Giorgini et al., 2023; Jones et al., 2024). Cultural practices—including reflective mulches, trap cropping, removal of weed hosts, and optimized planting dates—also reduce whitefly colonization pressure (Fonsah et al., 2018; Oliveira et al., 2001). Push–pull strategies, combining repellents (push) and attractive trap plants (pull), have further shown promise in reducing vector landings and subsequent virus transmission in vegetable systems (Lee et al., 2019). However, the rapid evolution of resistance (Bass et al., 2015), environmental concerns, and increasingly stringent regulatory frameworks (EU Directive 2009/128/EC, 2009) have undermined the sustainability of chemical control. Compounding this challenge, climate disruption has extended whitefly activity into previously unaffected regions and increased the number of reproductive cycles per season. As a result, host plant resistance breeding has become a crucial pillar of whitefly management, to offer season-long protection without environmental residue.

## Breeding for host resistance in vegetables

**Figure 1:**
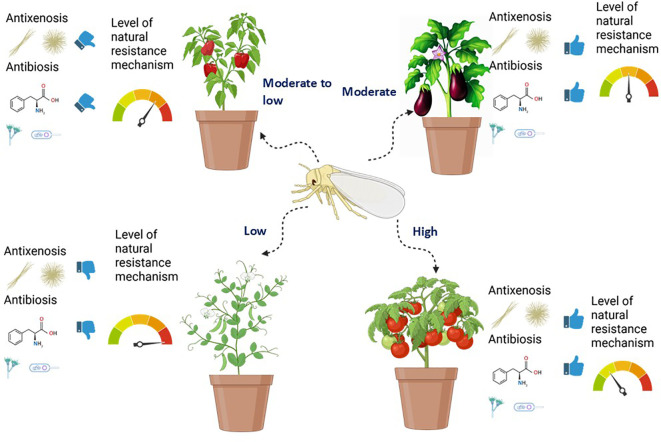
Schematic representation of level of antixenosis, antibiosis, and innate tolerance mechanisms in plants.

Even though breeding vegetables for resistance to whitefly-transmitted viruses is much needed, this review focuses on breeding for resistance to whiteflies. Although this review centers on whitefly resistance rather than virus resistance per se, it is important to note that traits conferring antibiosis or antixenosis against adult *Bemisia tabaci* often indirectly reduce the probability of primary and secondary virus spread (Kogan and Ortman, 1978; Raval and Trivedi, 2017). Reduced settling, shorter probing duration, and increased adult mortality directly limit the acquisition and inoculation efficiency of persistent–circulative begomoviruses, creating functional overlap between whitefly resistance and virus-transmission resistance (Legarrea et al., 2015; Marchant et al., 2020; Navas-Castillo et al., 2011). Plant breeding for host resistance has become an indispensable component, promising reduced chemical dependency, improved sustainability, and enhanced food security (Firdaus et al., 2013; Escobar-Bravo et al., 2016). Recent studies have highlighted the effectiveness of breeding programs in developing whitefly-resistant vegetable cultivars, emphasizing the role of genetic resistance in sustainable agriculture (Raval and Trivedi, 2017). Plants have evolved various resistance strategies to mitigate insect damage, broadly classified into three categories: antixenosis (nonpreference, repellence or deterrence of pest settling and feeding), antibiosis (negative impacts on pest survival and reproduction), and tolerance (the plant’s ability to withstand pest damage without significant yield losses) (Painter, 1951; Kogan and Ortman, 1978; Smith, 2005; Sulistyo and Inayati, 2016). However, resistance identified under controlled conditions does not always translate predictably to open-field environments, where temperature, humidity, host abundance, vector pressure, and mixed-virus infections influence expression of antixenosis and antibiosis. Several studies report genotype × environment interactions in which moderate resistance collapses under high vector pressure or under abiotic stresses such as heat or salinity, the latter recently shown to suppress whitefly resistance in *Capsicum* accessions (Caarls et al., 2025). These disparities highlight the need for multi-location validation of resistance sources. **Figure 1** is presented as a conceptual illustration to summarize general differences in inherent whitefly resistance observed across the major vegetable crops—where tomato typically exhibits the strongest combination of antixenosis and antibiosis, followed by eggplant and pepper, while snapbean often shows minimal inherent defense. This schematic representation highlights how host plant defenses function synergistically to deter infestation, impede whitefly growth, and reduce yield losses, providing the foundation for resistance breeding strategies in members of Solanaceae, Fabaceae, and other crops.

Early investigations into the resistance mechanisms highlighted the role that leaf morphological traits, such as trichome density and leaf thickness, can provide in conferring antixenosis and antibiosis, particularly in eggplant cultivars (Khan and Khan, 2018). Early studies in tomato identified the *Mi-1.2* gene as a key factor in antibiosis, affecting whitefly survival and reproduction (Nombela et al., 2003). In chili pepper, certain genotypes have been shown to exhibit antixenosis toward whiteflies, partly mediated by leaf surface traits and associated allelochemicals such as phenolics, flavonoids, and possibly terpenoids, which deter whitefly settling and oviposition (Firdaus et al., 2011; Hernández-Alvarado et al., 2019; Caarls et al., 2025). Furthermore, investigations into snapbean revealed that some cultivars possess tolerance mechanisms, maintaining yield despite whitefly presence, potentially linked to physiological adaptations Omae, 2005; UGA, 2021). Foundational studies across tomato, pepper, eggplant, and snapbean have greatly advanced understanding of host resistance mechanisms against whiteflies, though resistance often remains polygenic and linked to undesirable agronomic traits such as yield or fruit quality (Dias et al., 2016; Escobar-Bravo et al., 2016; Taher et al., 2020). An additional consideration is the evolutionary capacity of *B. tabaci*. Whiteflies can rapidly adapt to resistance traits through behavioral shifts, detoxification responses, biotype replacement, or symbiont-mediated plasticity. Deploying single resistance mechanisms may therefore impose selective pressure favoring more virulent biotypes, as observed in several regions following intensive use of partially resistant cultivars (Li et al., 2023). Ecological interactions—including natural enemy suppression, weed hosts acting as reservoirs, and climate-driven changes in whitefly phenology—further shape resistance durability (Navas-Castillo et al., 2011).

This review consolidates six decades of breeding progress in whitefly-resistant vegetables, highlighting key advances through marker-assisted selection, genomic selection, CRISPR/Cas9, speed breeding, and emerging AI-driven, high-throughput phenotyping, and multiomics approaches (Thabuis et al., 2004; Cappetta et al., 2021; Yoon et al., 2020; Watson et al., 2018; Chang et al., 2021; Song et al., 2022).

Recent studies have emphasized the epigenomic layer of defense regulation, where DNA methylation, histone modifications, and small RNAs dynamically modulate stress- and defense-responsive genes. In tomato, integrative methylome and transcriptome analyses during TYLCV infection revealed targeted differentially methylated regions linked to immune and signaling pathways, suggesting that epigenome editing and methylation-based priming could complement conventional resistance breeding (Romero-Rodríguez et al., 2023).

Despite their promise, these technologies face notable constraints. MAS and GS require dense marker–trait associations and large training populations, which are often unavailable for minor vegetables. CRISPR editing is constrained by regulatory barriers, off-target risks, and incomplete knowledge of pest–plant molecular interactions. Multiomics platforms generate high-dimensional datasets that demand significant computational capacity and bioinformatic expertise. AI and HTP approaches remain limited by annotation costs, hardware expense, and the need for robust models capable of generalizing across genotypes, environments, and infestation intensities. These limitations must be acknowledged when considering scalability for breeding programs.

## Insect-host interactions: current understanding and knowledge gaps

Whitefly–host interactions are shaped by the polyphagy and adaptability of *Bemisia tabaci* and *Trialeurodes vaporariorum*, the two major species affecting vegetable crops worldwide (De Barro et al., 2011; Gautam et al., 2022). *B. tabaci* comprises more than 40 cryptic lineages, with MEAM1 and MED being the most invasive and resistant, while *T. vaporariorum* is predominant in greenhouse systems of temperate regions (Karatolos et al., 2010). In key vegetable crops such as tomato and pepper, feeding by whiteflies causes chlorosis, sooty mold development due to honeydew accumulation, which reduce photosynthetic efficiency and marketability (Nombela et al., 2003). Host preferences vary by species and region: *B. tabaci* is predominant in tropical and subtropical regions and commonly affects open-field crops such as tomato, pepper, and eggplant, while *T. vaporariorum* is more frequently found in temperate climates and protected environments like greenhouses, where it infests crops such as cucumber (Jones, 2003; Karatolos et al., 2010). More critically, both species serve as vectors for over 300 plant viruses, most notably the begomoviruses, which include TYLCV in tomato and chili leaf curl virus (ChiLCV) or pepper yellow leaf curl virus (PepYLCV) in pepper (Seal et al., 2006; Rajagopalan et al., 2012; Thabuis et al., 2019).

In tomato, antixenosis is strongly associated with the presence of type IV glandular trichomes and acylsugar exudation, which deter whitefly settling, feeding, and oviposition by creating a sticky and inhospitable leaf surface (Firdaus et al., 2013; Lucatti et al., 2014; de Oliveira et al., 2023). Antibiosis involves the action of defensive secondary metabolites—primarily acylsugars, methyl ketones, and terpenoids—which negatively impact whitefly survival and fecundity. These compounds are often found in high concentrations in wild relatives like *S. habrochaites*. Introgressions from *S. habrochaites* into *S. lycopersicum* have been shown to confer resistance by enhancing type IV trichome density, increasing acylsugar biosynthesis (notably acylsucroses and acylglucoses), and modulating jasmonic acid–responsive genes, which collectively reduce *B. tabaci* survival and limit the efficiency of whitefly-transmitted virus (WTV) acquisition and transmission (Alba et al., 2009; Escobar-Bravo et al., 2016; Leckie et al., 2016). Field trials confirm that reductions in whitefly settling or probing—central behaviors targeted by antixenosis—translate into significantly lower primary infection rates of persistent begomoviruses. In tomato, acylsugar-mediated antixenosis has been associated with 40–60% reductions in TYLCV incidence under open-field conditions, while antibiosis-driven mortality can lower secondary spread by shortening the infectious window of viruliferous adults (Smeda et al., 2023). Quantitatively, introgressions from *S. habrochaites* have been reported to reduce adult whitefly survival by 30–70%, oviposition by 50–80%, and virus incidence by up to 60% across multi-location trials, demonstrating that laboratory-identified traits can confer meaningful epidemiological benefits in production systems (Marchant et al., 2020). Abiotic stresses such as heat, drought, or salinity can alter the metabolic pathways underlying resistance. For example, salt stress has been shown to reduce JA accumulation, weaken antibiosis, and suppress glandular trichome development in *Capsicum*, resulting in increased whitefly survival and oviposition (Caarls et al., 2025). Such environment-driven shifts explain why resistance expressed in controlled trials may be attenuated in field environments exposed to climatic extremes. Tolerance, though less characterized, is observed in some snapbean genotypes, where plants maintain acceptable yield under infestation, likely due to compensatory physiological responses or virus tolerance mechanisms (Agarwal et al., 2021). Whitefly performance is also shaped by ecological interactions beyond the plant.

However, several knowledge gaps still remain. The molecular basis of resistance, particularly tolerance, is not fully elucidated and may involve complex hormonal regulation and signaling pathways (Romero-Rodríguez et al., 2023). Resistance is often biotype-specific; thus, effectiveness against one *B. tabaci* lineage may not translate to another (Boykin et al., 2013). Environmental factors such as temperature and humidity also modulate resistance expression, complicating breeding for stable performance across diverse agroecologies (Dinsdale et al., 2010; Lucatti et al., 2013). Progress is further constrained by incomplete reference genomes for many vegetables, limited multi-biotype whitefly colonies, and inadequate high-throughput phenotyping tools for subtle behavioral traits. Coordinated sharing of biotype colonies, standardized EPG–imaging pipelines, and generation of pangenomes for pepper, eggplant, and snapbean would accelerate discovery. Durability of resistance also remains uncertain, as whiteflies can evolve counter-adaptations via biotype shifts, new endosymbionts, or behavioral avoidance. Documented resistance breakdowns highlight the need to pyramid multiple mechanisms—combining antixenosis, antibiosis, and virus-transmission interference—to delay evolutionary escape.

Whitefly performance is also shaped by ecological interactions beyond the plant. Natural enemies such as *Encarsia formosa*, predatory mites, and lady beetles can amplify host resistance, while microbial symbionts in *B. tabaci*—including *Hamiltonella*, *Rickettsia*, and *Wolbachia*—modulate fecundity, virus transmission efficiency, and plant defense responses (Tan and Hu, 2016). Endophytic fungi and bacteria may further enhance induced resistance or modify plant volatiles that affect whitefly attraction and feeding behavior (Aravinthraju et al., 2024). These insights form the foundation for deploying modern tools such as genomic selection and CRISPR to breed cultivars with enhanced, durable, and broad-spectrum resistance.

**Figure 2:**
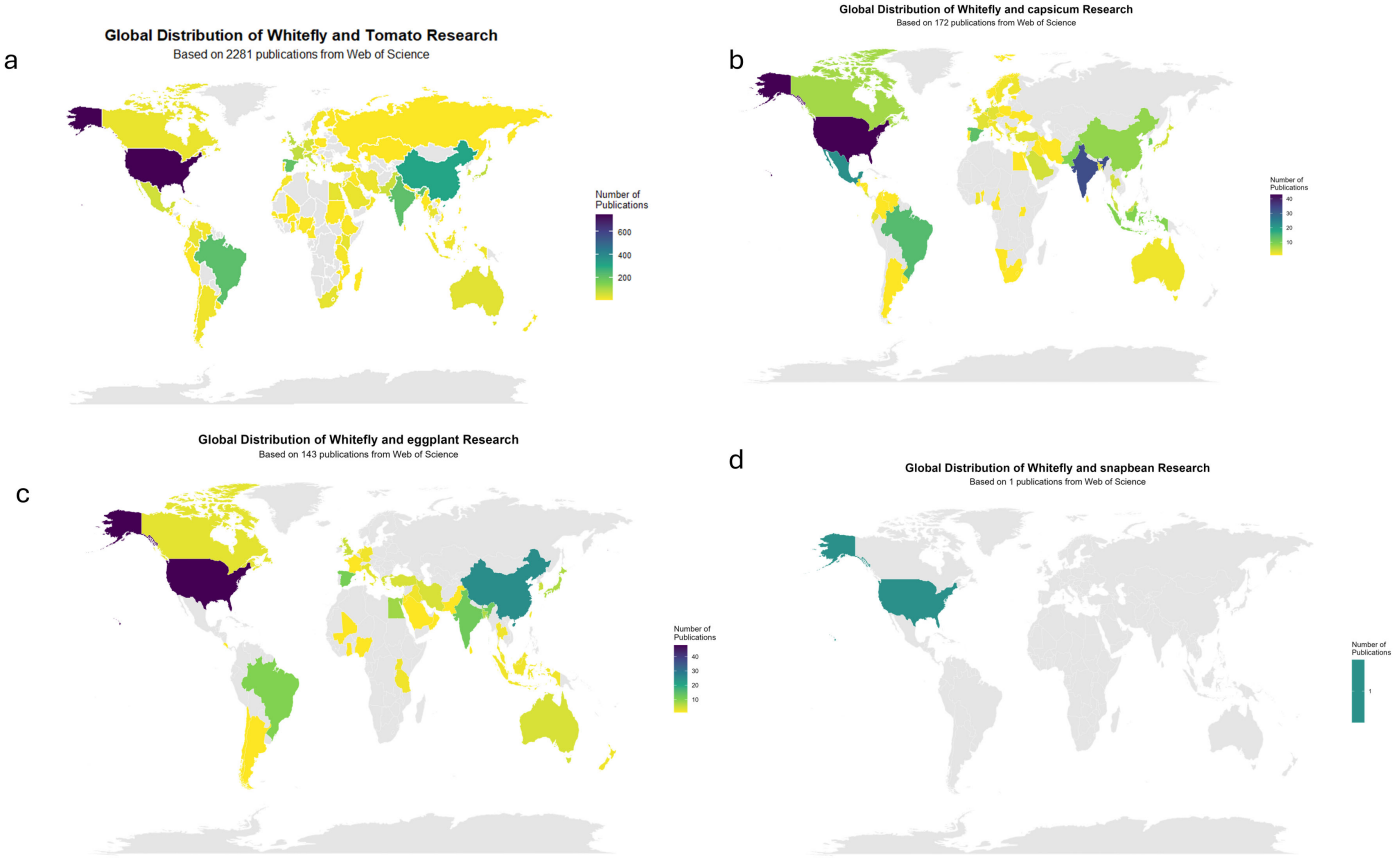
Global map showing number of publications on whitefly research in **(a)** Tomato **(b)** Capsicum **(c)** Eggplant **(d)** Snapbean.

**Figure 3:**
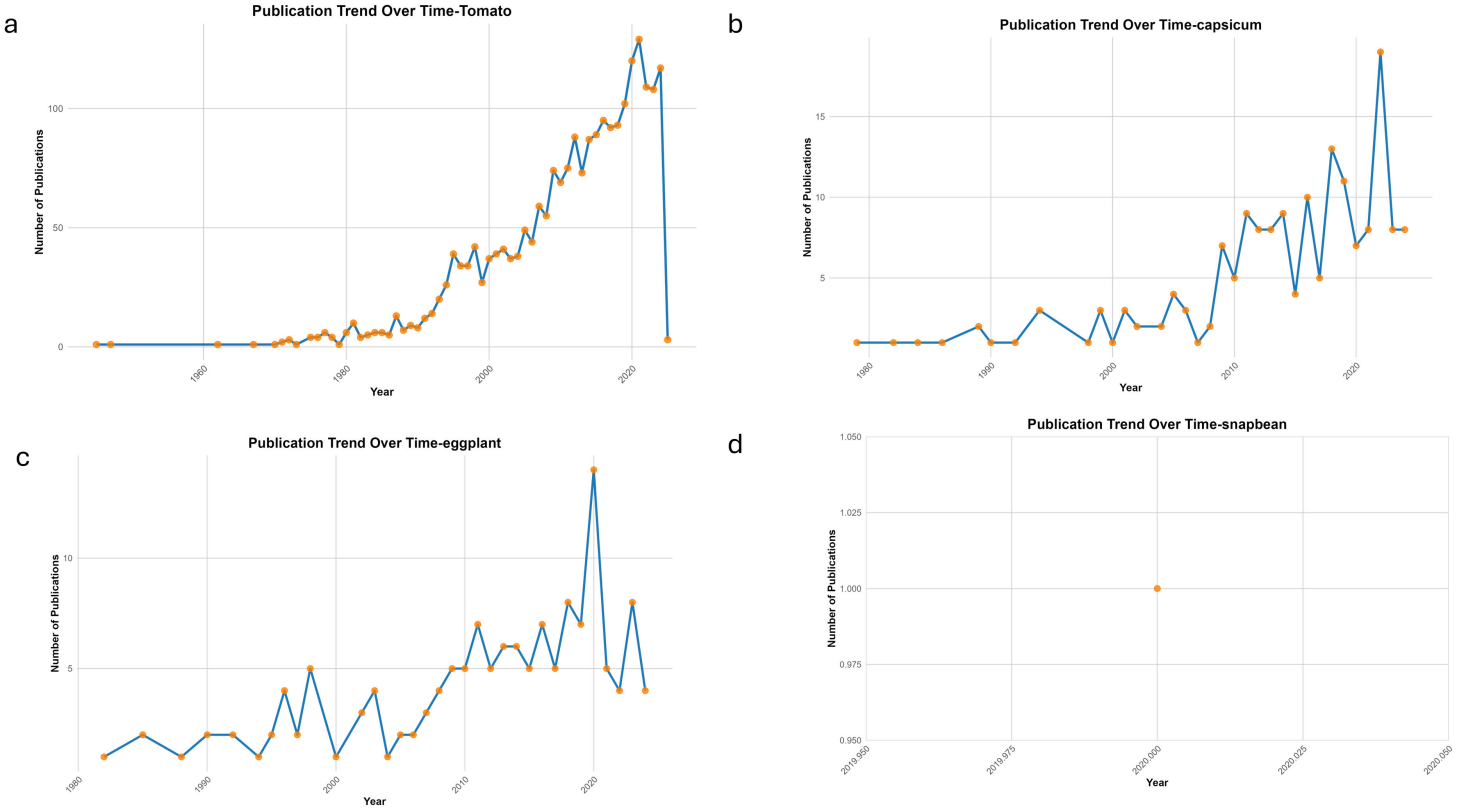
Publication trend on whitefly research in **(a)** Tomato **(b)** Capsicum **(c)** Eggplant **(d)** Snapbean.

Research activity on whitefly resistance has risen markedly in recent decades but remains uneven across crops and regions. Tomato dominates global research due to its economic importance and susceptibility to TYLCV, while pepper studies are regionally concentrated in Asia and Latin America. Eggplant and snapbean remain underrepresented despite their relevance in South Asia, sub-Saharan Africa, and the Americas. Publication trends (**Figures 2** and **Figure 3**) reveal imbalances tied to research capacity and funding: areas with the highest whitefly pressure and begomovirus incidence—such as South Asia, East Africa, and parts of Latin America—show limited output on these crops. This uneven scientific visibility underscores the need for targeted resource allocation to improve characterization and breeding of resistant cultivars in vulnerable production regions.

## Evolution of breeding strategies: from traditional to advanced techniques

### Conventional breeding

Conventional breeding has previously laid the groundwork for developing whitefly-resistant cultivars in vegetable crops. In tomato (*S. lycopersicum*), wild relatives such as *S. habrochaites*, *S. pennellii*, and *S. pimpinellifolium* have served as critical reservoirs of resistance traits (Tripathi and Varma, 2003). The wild species possess glandular type IV trichomes that secrete acylsugars—compounds that deter whitefly colonization and feeding by antixenosis and antibiosis mechanisms (Maliepaard et al., 1995; Marchant et al., 2020). Several breeding programs have successfully introgressed these traits into elite tomato backgrounds, although linkage drag and reduced fruit quality often accompany these transfers (Escobar-Bravo et al., 2016; Dias et al., 2016). In pepper (*C. annuum*), resistance sources have been identified in *C. annuum*, *C. chinense* and *C. frutescens*, which exhibit high trichome densities and accumulation of deterrent secondary metabolites capsaicinoids, phenolics, and flavonoids, which contribute to whitefly resistance through antixenotic effects (Kumar et al., 2006; Sandra and Maharijaya, 2022; Rini et al., 2025). However, the incorporation of these traits into commercial cultivars remains limited due to hybridization barriers and undesirable agronomic traits (Manzur et al., 2015; Shiragaki et al., 2022). In eggplant (*S. melongena*), wild relatives such as *S. torvum*, *S. incanum*, and *S. viarum* exhibit moderate levels of resistance, largely through trichome- and cuticle-based defenses. Wild accessions have been evaluated in segregating populations, but resistance breeding is still hampered by poor compatibility and limited molecular tools (Taher et al., 2020). In snapbean (*P. vulgaris*), resistance breeding efforts have primarily focused on virus resistance (Porch et al., 2013; Gepts et al., 2022). However, wild relatives like *P. acutifolius* show promise for direct whitefly resistance, particularly under abiotic stress. Few breeding programs have fully capitalized on this potential, reflecting the need for expanded germplasm screening and trait dissection (Agarwal et al., 2021). Overall, conventional breeding remains foundational but is hindered by polygenic inheritance, linkage drag, limited trait reproducibility- particularly in traits like trichome-based resistance which are rare and poorly validated across accessions. These limitations underscore the need for integrated molecular and genomic approaches to accelerate progress. To consolidate the current understanding of resistance mechanisms and their genetic basis across key vegetable crops, we summarized the major resistance genes, QTLs, and associated mechanisms reported in tomato, pepper, eggplant, and snapbean (**Table 1**), highlighting the diversity of resistance sources exploited so far and emphasizes existing gaps, particularly for under-studied crops. Beyond their direct effects on vector behavior, several of these resistance traits also influence virus transmission efficiency and epidemic development. **Table 2** summarizes representative studies demonstrating how whitefly resistance mechanisms translate into reduced virus acquisition, inoculation, and field-level disease incidence.

**Table 1:** Whitefly resistance mechanisms in key vegetable crops.

Crop	Gene/QTL/Mechanism	Function/Mechanism	Source/Origin	References
Tomato	Mi-1.2	Resistance to whitefly, nematodes, aphids via R-gene-mediated response	*Solanum peruvianum*	Nombela et al., 2003
Tomato	Acylsugar QTLs (AS)	Antixenosis and antibiosis through acylsugar-mediated deterrence	*S. pennellii*, *S. habrochaites*	Firdaus et al., 2013; Lucatti et al., 2013
Tomato	Wf-1 and Wf-2	QTLs associated with reduced whitefly survival and oviposition	*S. galapagense*	Firdaus et al., 2013
Tomato	Trichome IV Density QTL	Physical deterrence via increased glandular trichome density	*S. pimpinellifolium*	Mata-Nicolás et al., 2021
Tomato	Dual resistance mechanisms	Pre-phloem and phloem-located factors contributing to resistance	*L. pimpinellifolium*	Bleeker et al., 2016
Tomato	Non-trichome-based QTL	Resistance independent of glandular trichomes	*S. galapagense*	Lucatti et al., 2013; Santegoeds et al., 2021
Pepper	Whitefly resistance traits	Reduced oviposition and nymph development	*Capsicum annuum*	Firdaus et al., 2011
Pepper	Whitefly resistance traits	Reduced adult survival and nymph development	*Capsicum annuum*	Rini et al., 2025
Eggplant	Trichome QTLs	Physical deterrence via increased trichome density	*S. torvum*, *S. incanum*	Taher et al., 2020
Eggplant	Morphological and biochemical traits	Traits like leaf hair density and nitrogen content contributing to resistance	*S. melongena*	Khan and Khan, 2018
Snapbean	Cultivar-based resistance	Reduced whitefly oviposition and nymph development in specific cultivars	*Phaseolus vulgaris*	Li et al., 2022
Snapbean	Cultivar-based resistance	Not reported	*Phaseolus vulgaris*	Agarwal et al., 2021

**Table 2:** Summary of functional overlap between whitefly resistance and begomovirus transmission suppression.

Crop	Experimental focus	Observed epidemiological effect	Key insights	Reference	
Tomato–TYLCV	Field trials with acylsugar‐rich lines	40–60% lower TYLCV incidence and delayed symptom onset	Antixenosis limits virus acquisition and secondary spread	Escobar-Bravo et al., 2016; Legarrea et al., 2015
Tomato–TYLCV	Trichome IV-dense introgression lines	Fewer probes/shorter feeding; reduced virion uptake	Physical barriers complement chemical deterrence	Firdaus et al., 2013; Mata-Nicolás et al., 2021
Pepper–PepYLCV	High-phenolic/flavonoid genotypes	Decreased fecundity and virus accumulation	Metabolite-driven antibiosis lowers vector competence	Hernández-Alvarado et al., 2019; Sandra et al., 2022
Tomato (Ty gene pyramids)	Ty-1 + Ty-3 ± Ty-2 lines under natural infection	10²–10⁵-fold drop in viral DNA titers; near-absence of symptoms	Gene stacking provides additive suppression of begomoviruses	Gill et al., 2019; Hanson et al., 2016
Cross-crop comparisons	Multiple vegetables, flavonoid/terpenoid traits	Lower inoculation rates in brief probes (non-persistent viruses)	Surface chemistry influences short-term transmission efficiency	Li et al., 2023; Navas-Castillo et al., 2011

### Marker-assisted selection and genomic selection

Marker-assisted selection (MAS) has proven effective in identifying and introgressing resistance loci into elite cultivars. In tomato, MAS has been used to transfer QTLs linked to acylsugar biosynthesis and type IV trichome development from wild species such as *S. galapagense* and *S. habrochaites* (Sajjanar and Balikai, 2009; Firdaus et al., 2013). These tools have also supported the pyramiding of viral resistance genes/alleles, including Ty-1, Ty-2, Ty-3, Ty-4, ty-5, and Ty-6, which indirectly mitigate whitefly damage by limiting virus replication and movement (Zamir et al., 1994; Ji et al., 2009; Lapidot et al., 2015; Gill et al., 2019). In pepper, MAS has primarily targeted resistance to viral (*PepYLCV*) and fungal pathogens (*Phytophthora capsici*) (Srivastava et al., 2017; Thakur et al., 2019); however, synteny with tomato genomic regions harboring whitefly resistance QTLs suggests the potential to extend MAS to insect resistance traits (Thabuis et al., 2004). Eggplant breeding programs have mapped QTLs linked to trichome density and pest deterrence, but validated molecular markers are still scarce (Frary et al., 2003). Genomic selection (GS) builds upon MAS by using genome-wide markers to predict complex phenotypes, offering a powerful approach to manage polygenic resistance traits. GS has been effectively applied to complex traits in tomato, such as abiotic stress tolerance, for instance GS models have demonstrated high prediction accuracies for yield and soluble solid content under heat stress conditions, highlighting the potential of GS in enhancing stress resilience in tomato breeding programs (Cappetta et al., 2021). However, GS models specifically targeting acylsugar-mediated whitefly resistance in tomato have not yet been reported. The successful applications of GS in complex trait improvement suggest its promising utility in accelerating the development of whitefly-resistant tomato cultivars by enabling the prediction of breeding values for resistance traits based on genome-wide marker data. In pepper (*Capsicum* spp.), GS models have been developed to predict fruit-related traits, demonstrating the potential of GS in accelerating breeding for quality attributes (Hong et al., 2020). For eggplant (*S. melongena*), while direct GS studies are limited, the availability of high-quality genome assemblies and the development of genomic resources provide a solid foundation for future GS applications (Gramazio et al., 2019; Yu et al., 2024a). In snapbean (*P. vulgaris*), GS has been utilized to predict agronomic traits under various environmental conditions, indicating its utility in improving complex traits in this crop (Keller et al., 2020). These advancements emphasize the expanding role of GS in vegetable crop improvement, offering a framework for its application in breeding programs targeting traits such as whitefly resistance. While MAS has already delivered impact in tomato and pepper, the integration of GS promises broader gains across these crops, especially in accelerating multi-trait improvement under whitefly pressure.

### Dual strategy for whitefly management in tomato: gene pyramiding and insect resistance from wild relatives

Efforts to mitigate the devastating impact of whitefly-transmitted viruses in tomato have traditionally focused on breeding for virus resistance. However, breeding programs have adopted a complementary dual strategy targeting both the vector and the virus it transmits, particularly Tomato Yellow Leaf Curl Virus (TYLCV) and related begomoviruses such as Tomato yellow leaf curl Thailand virus (TYLCTHV). This integrated approach combines the use of gene pyramiding to enhance virus resistance and the deployment of insect resistance genes derived from wild tomato relatives to reduce whitefly population pressure and virus transmission. Pyramiding resistance genes (Ty-1, Ty-2, Ty-3, and Ty-6) has proven an effective method for achieving durable resistance against begomoviruses (Anbinder et al., 2009; Lapidot et al., 2015). In ongoing breeding programs, we have developed and field-tested a series of advanced lines and hybrids carrying different combinations of these Ty genes. Through both MAS and field phenotyping, we demonstrated that specific gene combinations, particularly Ty-1 with Ty-3 or Ty-6, confer enhanced resistance to TYLCV and TYLCTHV. However, pyramiding multiple Ty genes is not without trade-offs. Several studies have reported that introgressions from wild relatives can sometimes be linked with reduced yield, delayed maturity, altered fruit shape, or changes in firmness and flavor, particularly when large genomic segments are transferred (García-Martínez et al., 2014). Recent evaluations of Ty-1 and ty-5 introgression into traditional ‘De la pera’ backgrounds, for example, showed that while TYLCV tolerance was maintained, some lines exhibited modifications in yield components and fruit quality attributes that required additional selection to recover market-preferred phenotypes (García-Martínez et al., 2016). These observations highlight the importance of carefully monitoring agronomic performance and quality traits when stacking resistance genes.

Recent findings show that the accumulation of TYLCV in tomato tissues is significantly reduced in lines pyramided with two or more Ty genes, compared to monogenic carriers. In pyramided hybrids carrying Ty-1/Ty-3 together with Ty-2, viral DNA levels have been reported to drop by two to five orders of magnitude compared to susceptible lines, with some cultivars showing 10²–10⁵-fold lower TYLCV or TYLCSV accumulation at several weeks post-inoculation and complete absence of visible symptoms (Tabein et al., 2017). Field evaluations of lines combining Ty-2 and Ty-3 have likewise documented substantial reductions in disease severity across multiple begomovirus species (Lee et al., 2020). Such quantitative reductions in virus load provide a strong empirical basis for deploying pyramided Ty genotypes in high-pressure environments. These results were validated under open-field conditions with high natural inoculum pressure. The additive or possibly synergistic effects of Ty gene combinations provide a more robust barrier against emerging virus strains, emphasizing the value of multigenic resistance in breeding strategies (Hanson et al., 2016). In parallel, tomato lines exhibiting strong resistance to the whitefly vector (*B*. *tabaci*) itself, using *S*. *galapagense* as the resistance donor were developed (Firdaus et al., 2012). This wild species reduced whitefly feeding and oviposition, delayed development, and lower adult emergence. Our current work further explores the interaction between insect resistance traits and viral accumulation. The data suggest that combining insect resistance with Ty gene pyramiding may provide a dual-layered defense system, one that limits both initial infection and subsequent virus spread in the field. A critical question is whether strong insect resistance always translates into reduced virus transmission. While acylsugar- and trichome-based resistance from *S. galapagense* can substantially depress whitefly survival, feeding, and oviposition, these traits also impose metabolic costs on the plant and may interact with other resistance pathways (Vendemiatti et al., 2021). In some backgrounds, high acylsugar levels have been associated with increased leaf stickiness, altered canopy microclimate, or slight yield penalties, raising the possibility that multiple, concurrently expressed defense pathways might reduce overall plant vigor under certain conditions (Marchant et al., 2020). Carefully designed field studies that track both whitefly behavior and virus incidence, alongside yield and quality, are therefore essential to determine when insect resistance and Ty pyramiding are complementary and when trade-offs emerge. The performance of Ty pyramids is also strongly influenced by the genetic background and the composition of local virus populations. Lines carrying identical Ty gene combinations can exhibit different resistance levels when introgressed into determinate vs indeterminate backgrounds or traditional vs modern cultivars, reflecting epistatic interactions and linkage drag (Scott et al., 2015). Likewise, studies from Asia and the Mediterranean region have shown that some pyramids are highly effective against certain monopartite TYLCV isolates but less so against specific bipartite or recombinant begomoviruses, underscoring the necessity of tailoring pyramiding strategies to regional virus diversity (Kesumawati et al., 2020). The convergence of virus resistance and vector resistance within a single breeding program represents a paradigm shift toward more sustainable whitefly management. Such integration enhances the durability of resistance, reduces selection pressure on the virus and the insect, and aligns well with climate-resilient breeding frameworks (Navas-Castillo et al., 2011). At the same time, multigenic resistance does not eliminate evolutionary risk. Local begomovirus populations can be highly diverse, and several reports show that certain Ty gene combinations, or individual Ty genes, are insufficient against new or mixed infections of distinct local strains, indicating partial breakdown or leakage of resistance (Koeda et al., 2020). Similarly, shifts in *B. tabaci* species composition or biotype prevalence may alter vector competence and interact differently with host resistance. Consequently, pyramided lines should be evaluated regularly against the evolving virus complex and vector populations in target regions, and new resistance sources should be continuously incorporated to avoid over-reliance on a limited Ty gene set. Overall, Ty pyramiding and vector resistance from wild relatives provide powerful tools, but their deployment must explicitly account for trade-offs in yield and quality, background-specific effects, and the evolutionary responses of both viruses and whitefly populations if durability is to be achieved.

### CRISPR/Cas9 gene editing

CRISPR/Cas9 genome editing has ushered in a new era of precision breeding for complex traits such as whitefly resistance. In tomato, targeted editing of susceptibility genes like eukaryotic translation initiation factor 4E1 *(eIF4E1)* has conferred resistance to whitefly-transmitted viruses including Pepper mottle virus (PepMoV), while maintaining favorable agronomic traits (Yoon et al., 2020). Biologically, eIF4E1 functions as a cap-binding translation initiation factor that many RNA viruses, including potyviruses, hijack via their VPg or related proteins to translate viral RNAs and complete their replication cycle (Moury et al., 2014). Loss-of-function or allele modifications in eIF4E1 disrupt this interaction, thereby blocking efficient viral protein synthesis and systemic movement. Although such edits do not directly alter whitefly feeding behavior, the strong reduction in viral titer in plant tissues is expected to lower acquisition efficiency by vectors and diminish subsequent transmission (Piron et al., 2010). In edited tomato lines, eIF4E1 modifications have been associated with drastic reductions in virus accumulation and symptom expression, with many inoculated plants remaining either symptomless or showing only mild, transient symptoms compared with severe stunting and mosaic in wild-type controls (Yoon et al., 2020). High editing frequencies at the target locus and stable inheritance across generations indicate that such alleles can be efficiently fixed in breeding populations once suitable transformation pipelines are in place. Similarly, manipulation of defense-related genes such as *Mi-1.2*, an NBS-LRR resistance gene conferring broad-spectrum resistance to phloem-feeding pests including whiteflies, aphids, and nematodes, has demonstrated potential in enhancing plant immunity (Nombela et al., 2003). Tashkandi et al. (2018) successfully employed CRISPR/Cas9 to target the Tomato yellow leaf curl virus (TYLCV) genome via coat protein and replicase sequences, resulting in effective viral interference and reduced TYLCV DNA accumulation in *Nicotiana benthamiana* and tomato, with heritable resistance.

While CRISPR/Cas9 has yet to be applied directly for engineering whitefly resistance in tomato, the identification of resistance QTLs on chromosomes 9, 10, and 11 (Momotaz et al., 2010), as well as loci regulating glandular trichome density and acylsugar production (Escobar-Bravo et al., 2016), provides a roadmap for such interventions. Beyond targeting viral susceptibility genes, direct genome editing of insect-resistance pathways is an emerging frontier. In tomato, transcription factors such as Wo and SlMYC1 regulate glandular trichome initiation and type-IV trichome density—key traits underlying acylsugar-mediated whitefly deterrence (Yang et al., 2011; Galdon-Armero et al., 2018). Likewise, CRISPR modification of acylsugar acyltransferase genes (ASAT1–4) offers a strategy to fine-tune acylsugar profiles for optimal antixenosis and antibiosis (Nadakuduti et al., 2017). These targets exemplify how genome editing can move beyond viral defense to engineer direct morphological and metabolic barriers against whitefly infestation in vegetable crops.

It is also important to recognize that editing single susceptibility or defense genes often results in partial or strain-specific resistance (Wang et al., 2025). Given the quantitative and networked nature of whitefly and virus resistance, multiplex editing—simultaneously targeting several susceptibility factors, regulators of trichome development, or key nodes in JA/SA signaling—is likely to be necessary for durable, broad-spectrum outcomes. Network-level editing strategies that modulate regulatory hubs, rather than single effectors, may better reflect the polygenic architecture revealed by QTL and omics studies. Importantly, gene editing holds the promise to overcome historical challenges of linkage drag — where resistance traits introgressed from wild relatives are associated with unfavorable fruit size or quality — by enabling targeted disruption of deleterious genomic linkages while retaining beneficial loci. This represents a paradigm shift from traditional breeding, where such trade-offs have slowed cultivar development.

In practice, CRISPR is best viewed as complementary to, rather than a replacement for, conventional and molecular breeding. QTL mapping, GWAS, MAS, and genomic selection are essential to identify robust resistance loci and favorable haplotypes, while CRISPR offers a means to fine-tune those alleles, knock out residual susceptibility genes, or break unfavorable linkages within already adapted backgrounds. Such integration allows breeders to stack resistance derived from wild relatives with precisely edited susceptibility alleles, building more realistic and scalable improvement pipelines.

In pepper, editing of the susceptibility gene *CaMLO2* using CRISPR/Cas9 has demonstrated editing efficiencies of 6.3–17.7% across multiple Chile pepper cultivars, suggesting its broader utility in disease resistance improvement (Park and Kim, 2023). While functional genomic resources for eggplant remain limited, preliminary efforts have focused on developing genome-editing pipelines and identifying candidate genes for resistance breeding (Prajapati et al., 2023). In snapbean, direct CRISPR applications are currently lacking, but translational insights from soybean suggest feasible paths forward (Zhang et al., 2022). However, the efficiency of these pipelines remains highly genotype dependent, and many elite cultivars exhibit poor regeneration or transformation responses. This genotype specificity currently limits the direct deployment of CRISPR edits into farmer-preferred varieties in pepper, eggplant, and snapbean, and underscores the need for improved tissue-culture-independent delivery systems or broadly applicable transformation protocols. Despite its promise, CRISPR/Cas9 is constrained by several practical limitations. Transformation efficiency and plant regeneration remain major bottlenecks in many Solanaceae and Fabaceae genotypes, with strong cultivar dependence and frequent somaclonal variation (Yang et al., 2024). Editing efficiency can vary widely between targets and backgrounds, and off-target events, although reduced with newer nucleases, still require careful screening. In crops such as pepper, eggplant, and snapbean, robust genotype-independent transformation systems are not yet routine, which restricts CRISPR application to a narrow set of amenable lines and complicates direct editing of locally preferred cultivars. Translating CRISPR-derived resistance from proof-of-concept to commercial cultivars will also depend on biosafety regulation, consumer acceptance, and intellectual property landscapes. Regulatory frameworks for gene-edited crops differ markedly between regions, with some jurisdictions treating certain edits similarly to conventional mutations and others applying GMO-like regulations. Public perception of genome-edited vegetables and the costs of navigating overlapping IP on Cas nucleases, guide RNA architectures, and transformation technologies can influence whether CRISPR-based whitefly- and virus-resistant varieties reach farmers, especially in low- and middle-income countries. Looking ahead, multiplex editing, base editing, and gene regulation platforms offer scalable avenues for stacking resistance loci, breaking unfavorable linkages, and accelerating the development of durable, high-yielding, whitefly-resistant cultivars. Altogether, CRISPR/Cas9 offers powerful opportunities to engineer virus and vector resistance, but its impact will ultimately depend on overcoming transformation bottlenecks, integrating edits with existing breeding pipelines, and moving beyond single-gene targets toward multiplex, network-informed designs.

### Speed breeding

Speed breeding represents a powerful acceleration tool to fast-track the development of whitefly-resistant lines, particularly in crops with long generational cycles or complex trait integration needs. This process relies on controlled environmental conditions of extended photoperiods, temperature optimization, and regulated humidity to reduce generation time (Watson et al., 2018). In tomato, successful implementation of speed breeding protocols has enabled up to four generations per year significantly hastening the incorporation of resistance loci such as those linked to acylsugar production (Gimeno-Páez et al., 2025). Adaptations of this approach have also been established in pepper, where recent studies show reductions in time to flowering and fruiting by nearly 50% under LED-controlled chambers (Choi et al., 2023). Although still under development for eggplant and snapbean, preliminary studies suggest that light quality manipulation and early seed harvesting can significantly reduce generation intervals in these crops as well. Notably, efforts in eggplant using rapid cycling protocols have shown promise for integrating trichome-based resistance into elite lines (Frary et al., 2003). In legumes, protocols developed for chickpea and common bean offer a foundation for tailoring speed breeding in snapbean (Samantara et al., 2022). When combined with MAS, GS, or CRISPR/Cas technologies, speed breeding critically facilitates the pyramiding of resistance traits and the rapid advancement of segregating populations. The synergy of these approaches offers a pathway for responsive, climate-resilient whitefly resistance breeding programs.

**Figure 4:**
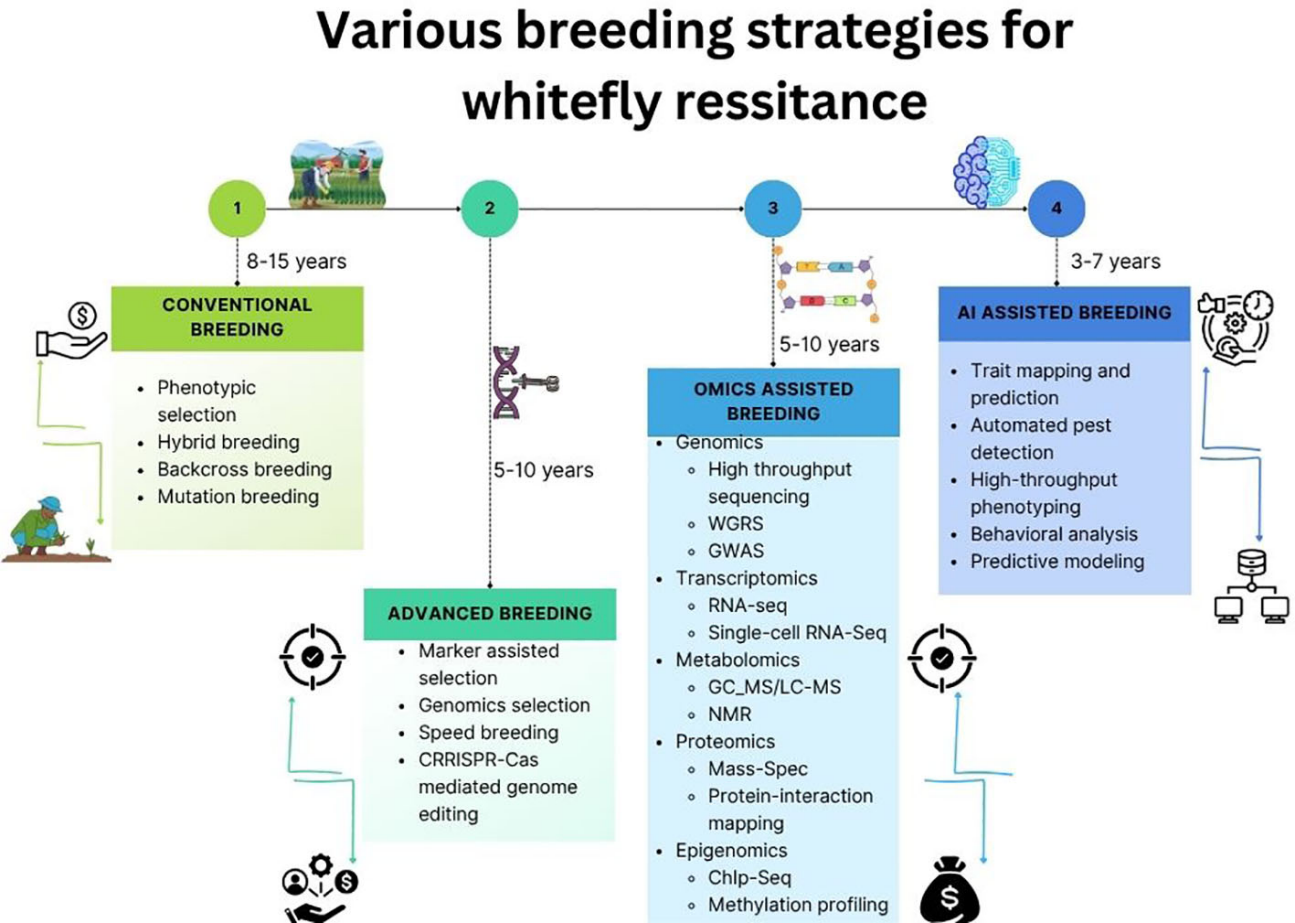
Various breeding strategies towards developing whitefly resistance in vegetable crops.

To encapsulate the progression from foundational breeding approaches to cutting-edge technologies, **Figure 4** illustrates the diverse strategies employed in developing whitefly-resistant vegetable cultivars. This visual highlight how classical methods like wild-relative introgression have evolved into genomics-driven breeding, gene editing, and AI-enabled predictive platforms, reflecting the dynamic shift towards precision and speed in resistance breeding.

## Emerging technologies transforming resistance breeding

### Artificial intelligence and high-throughput phenotyping

**Figure 5:**
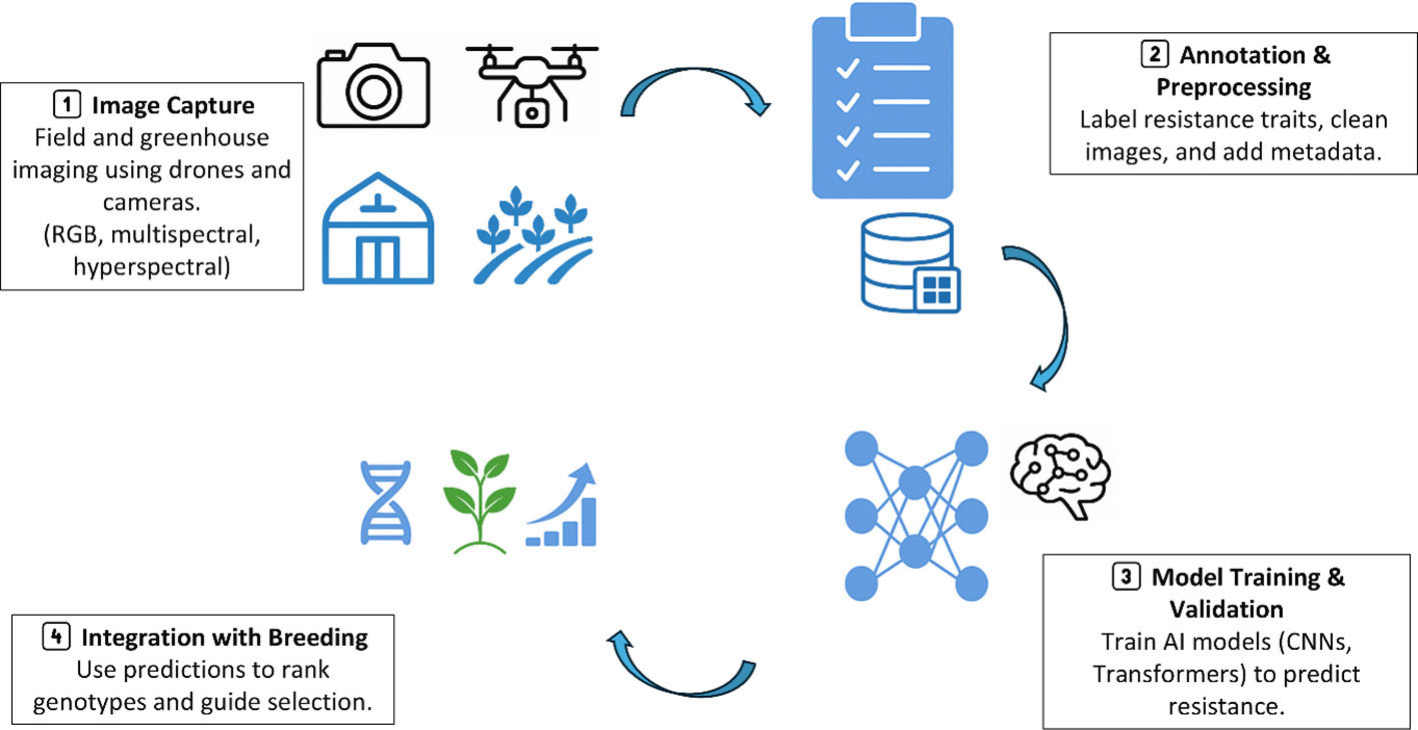
Schematic workflow illustrating the AI–HTP pipeline.

The integration of artificial intelligence (AI) and high-throughput phenotyping (HTP) is redefining resistance breeding by addressing one of its core bottlenecks: phenotyping accuracy and scalability (Jangra et al., 2021). The schematic workflow of AI-HTP pipeline is illustrated in **Figure 5**. Traditional phenotyping approaches are often subjective, time-consuming, and prone to human error. AI-powered imaging and sensor-based platforms provide a transformative alternative for assessing resistance-associated traits like whitefly-induced chlorosis, trichome density, and insect feeding scars across large breeding populations (Dolatabadian et al., 2025). In practice, however, AI-HTP platforms face several limitations. Acquisition and maintenance of imaging systems, drones, and sensors are costly, and require specialized technical staff. Many current tools are optimized for specific crops, canopy architectures, and imaging conditions, which limits their transferability to diverse agroecological settings. Moreover, the volume of raw data generated can easily exceed the data management and processing capacity of small and medium breeding programs.

Recent advancements in deep learning and imaging technologies have significantly enhanced the detection and monitoring of whitefly infestations in tomato crops. Nieuwenhuizen et al. (2018) developed a method using Faster R-CNN to detect whiteflies and their predatory bugs on yellow sticky traps in tomato crops. Their approach involved imaging the traps under controlled and uncontrolled conditions and achieved a weighted average accuracy of 87.4% for insect detection. Additionally, Abdulridha et al. (2020) utilized hyperspectral imaging combined with machine learning to identify and classify tomato yellow leaf curl virus (TYLCV) infections in tomato plants. While this study focused on viral infections rather than whitefly infestations, it highlights the applicability of hyperspectral imaging and machine learning in detecting plant stress symptoms. Devi et al. (2023) developed a novel automated tool for fast quantification of whitefly eggs on tomato leaves, using a deep learning-based object detection model was trained using the collected images, deployed in a web-based application called Eggsplorer. These studies emphasize the emerging role of deep learning and imaging technologies in plant health monitoring, though direct applications for whitefly detection on tomato plants using both RGB and hyperspectral images remain an area for future research.

Drone-mounted multispectral cameras and thermal sensors are also increasingly being deployed for large-scale field phenotyping, particularly in breeding trials targeting complex traits. While pepper and eggplant applications remain in early phases, progress is being made in adapting AI-HTP tools to capture morphological and physiological indicators of resistance under greenhouse and field conditions. These tools can also detect subtle changes in reflectance or temperature signatures indicative of early pest attack or stress response. The integration of AI with phenomics not only enables non-invasive, real-time assessment but also provides robust datasets that can inform genomic selection models, accelerate QTL discovery, and train machine learning models for trait prediction. Critically, these outputs must translate into actionable breeding decisions. AI-derived traits—such as predicted infestation intensity, chlorosis indices, or trichome density scores—can be used to rank lines, remove clearly susceptible genotypes early, and prioritize a smaller subset for deeper molecular or field validation (Abdulridha et al., 2020). When incorporated into genomic selection models, AI-HTP traits act as secondary phenotypes that improve prediction accuracy, directly influencing which families advance, which crosses are repeated, and how trial resources are allocated. Implementing these systems at scale requires robust data pipelines: standardized image acquisition protocols, reliable data transfer from field to server, secure storage, and automated preprocessing (Gong and Diao, 2025). Training deep learning models demands large, well-annotated datasets and computing infrastructure that may not be available in many public breeding programs. Field hardware—such as drones, fixed cameras, and sensors—must also be maintained, calibrated, and protected from environmental stress, adding recurrent costs beyond initial deployment.

However, standardization of phenotyping protocols and availability of annotated image datasets remain significant challenges that must be addressed through interdisciplinary collaboration between plant scientists, data engineers, and breeding programs. A major gap is the lack of standardized trait definitions, metadata formats, and benchmarking datasets across studies. This limits model comparability and reuse. Moving forward, shared metadata frameworks, common ontologies for whitefly-related traits, and open-source image repositories would greatly accelerate method development. Such standardization would also facilitate cross-location model validation and reduce duplication of effort. Another critical concern in AI-guided phenotyping is the potential for “escapes”—plants that appear uninfested not due to inherent resistance but because of uneven pest pressure or environmental shielding. AI-based phenotyping is vulnerable to multiple error sources, including misclassification of symptoms caused by nutrient deficiency, heat stress, or other pests; variation in illumination; occlusion by neighboring leaves; and shifts in model performance when applied across environments. To ensure biological accuracy, AI-derived measurements must be validated with complementary modalities such as qPCR-based virus quantification, phytohormone profiling, or EPG feeding assays, ensuring that predictions reflect true resistance rather than imaging artefacts. AI-enabled HTP platforms are poised to become indispensable components of next-generation whitefly resistance breeding pipelines, offering scalability, precision, and integration with digital breeding tools (**Figure 4**). As shown in **Figure 4**, AI-HTP integrates into multiple stages of a breeding pipeline. In early generations, it enables rapid removal of highly susceptible lines based on image-derived resistance traits. In intermediate phases, repeated AI-based measurements across environments serve as informative secondary phenotypes for genomic selection and QTL mapping. In later stages, AI-HTP facilitates stability assessments by quantifying how lines respond to variable pest pressure and abiotic stress. Thus, AI-HTP contributes not only to faster data collection but also to more informed, evidence-based breeding decisions.

### Multiomics integration

Multiomics approaches provide a comprehensive systems-level perspective of plant defense responses to whitefly infestation and are revolutionizing the identification and deployment of resistance traits. Whole-genome resequencing and RNA-seq analyses in tomato have uncovered regulatory networks activated in response to *B*. *tabaci* feeding and begomovirus infection, particularly involving the jasmonic acid (JA) and salicylic acid (SA) pathways (Song et al., 2022). Differential expression of transcription factors, receptor-like kinases, and hormone-regulating genes have also been observed in resistant versus susceptible lines (Escobar-Bravo et al., 2016). Further transcriptomic analysis revealed that feeding punctures by the predator *Nesidiocoris tenuis* induced the expression of defensive genes in the jasmonic acid signaling pathway in tomato plants with type IV trichomes, resulting in repellence to whiteflies and attractiveness to *N*. *tenuis* (Riahi et al., 2023). In pepper and eggplant, early transcriptomic studies have started to unravel candidate genes associated with virus tolerance and stress signaling (Góngora-Castillo et al., 2012; Zhou et al., 2016). Despite rapid progress, current transcriptomic datasets remain limited in genotype diversity, developmental stages, and environmental conditions. Many studies rely on controlled-environment assays, which do not fully recapitulate the fluctuating temperatures, mixed infections, or variable whitefly pressure experienced in the field. The absence of reference-quality pangenomes for pepper, eggplant, and snapbean restricts the ability to map structural variants or presence–absence variations underlying resistance. Moreover, interactions among plant, virus, and whitefly genotypes remain largely unexplored, highlighting the need for multi-environment, multi-genotype omics analyses to capture ecologically relevant defense responses.

Acylsugars, phenolic compounds, and terpenoids have been identified as key metabolites associated with whitefly resistance in tomato and pepper. Studies have shown that combining acylsugars with zingiberene results in a synergistic effect, further reducing whitefly fitness (de Oliveira et al., 2023). Targeted and untargeted metabolomic profiling has enabled the co-localization of metabolite signatures with resistance QTLs, facilitating marker development and trait dissection (Firdaus et al., 2013). For instance, resistant pepper genotypes exhibit enhanced accumulation of flavonoids and antioxidant enzymes post-infestation (Wu et al., 2019). A critical limitation of metabolomic studies is that metabolite profiles are highly plastic and strongly influenced by abiotic stress. Heat, drought, and salinity can suppress JA-dependent metabolite pathways or reduce glandular trichome development, leading to weakened resistance even in genetically resistant lines. Recent evidence in *Capsicum* shows that salt stress diminishes JA accumulation and increases whitefly survival (Caarls et al., 2025), underscoring the need to integrate abiotic × biotic stress metabolomics to understand how resistance traits perform under real-world conditions.

Although still nascent in vegetable crops, epigenetic regulation is emerging as a potential mechanism of induced resistance. In tomato, methylome and small RNA analyses under TYLCV infection have revealed dynamic DNA methylation changes and small RNA-mediated gene silencing as potential contributors to defense (Romero-Rodríguez et al., 2023). Such studies remain limited or unexplored in eggplant and snapbean, presenting an opportunity for future research. However, epigenomic datasets in vegetables are still sparse, and most studies examine single stresses rather than the combined pressures typical of agricultural systems. It remains unclear whether virus- or herbivory-induced methylation changes are stable enough to serve as heritable breeding targets, or whether they require repeat induction each generation. Integrating methylome, sRNA, and chromatin accessibility data will be essential to determine whether epigenetic signatures can be used as predictive biomarkers or engineered through epigenome-editing tools.

A recent study has revealed how viral infection modulates the proteome of whitefly vectors and their plant hosts. For instance, a comparative study showed that Tomato yellow leaf curl virus (TYLCV) differentially alters DNA methylation patterns in two cryptic species of *B*. *tabaci*, linking viral transmission competence to proteome and epigenome remodeling (Catto et al., 2023). While still nascent, phenomics and volatilomics are being explored through AI-based imaging and thermal sensing platforms to detect early pest-induced stress signatures in whitefly-infested crops. True multiomics integration remains challenging due to differences in sampling timelines, tissue specificity, data dimensionality, and normalization methods across omics platforms. Many studies analyze each omics layer independently, missing opportunities to construct causal models linking transcriptional regulation, metabolite accumulation, trichome morphology, and whitefly performance. There is an urgent need for standardized pipelines for cross-omics normalization, network inference, and data fusion, as well as publicly accessible integrative datasets spanning transcriptomics, metabolomics, and phenomics under harmonized conditions. Multiomics data integration holds immense potential for the identification of resistance biomarkers, prioritization of candidate genes, and development of predictive breeding models. Coupling these insights with AI and genome editing platforms offers a path toward highly targeted, knowledge-driven breeding strategies for durable whitefly resistance. Importantly, multiomics outputs directly inform breeding strategy by narrowing candidate gene lists, identifying metabolite markers for early-stage screening, and strengthening genomic selection models through biologically grounded priors. Integrating omics-derived biomarkers into MAS or GS pipelines can accelerate selection cycles, while network-level insights help identify regulatory hubs suitable for CRISPR multiplex editing. Thus, multiomics does more than describe plant responses—it guides which traits to target, which germplasm to select, and which edits or crosses are most likely to produce durable, field-relevant resistance.

### Genomic language models and AI-powered predictive breeding

Genomic language models (gLMs) represent a cutting-edge application of artificial intelligence that treats genomic sequences as structured linguistic data. These models are capable of learning contextual relationships between genetic elements, enabling functions such as gene annotation, regulatory motif identification, and variant effect prediction with unprecedented accuracy. In plant genomics, gLMs are increasingly being used to enhance trait discovery and accelerate breeding decisions. While their application in vegetable crops remains exploratory, early models trained on tomato and *Arabidopsis* genomes have shown promise in predicting promoter activity, guiding sgRNA design for CRISPR editing, and identifying non-coding regulatory elements linked to stress responses (Farooq et al., 2024; Huang et al., 2024; MacNish et al., 2025). Unlike classical genomic prediction models such as GBLUP, rrBLUP, or Bayesian regressions—which rely on marker matrices and assume linear or semi-linear effects—gLMs treat DNA sequences as structured language and learn regulatory grammar directly from sequence context (Tyagi et al., 2025). This allows the capture of long-range interactions, motif dependencies, and non-linear regulatory rules that traditional models overlook. Unlike CNNs or standard Transformers that operate on short genomic windows, plant foundation models such as AgroNT, PlantCaduceus, and PlantRNA-FM are pre-trained on billions of nucleotides from multiple plant genomes, allowing generalization across genomic contexts without manual feature engineering (Mendoza-Revilla et al., 2024; Yu et al., 2024b). A key limitation of current gLMs is species bias: because most training corpora derive from model genomes such as *Arabidopsis*, rice, or tomato, prediction accuracy declines when applied to distantly related vegetable crops with large, repetitive, or poorly annotated genomes such as pepper, eggplant, or snapbean. Transfer-learning and domain-adaptation strategies—where pre-trained models are fine-tuned using crop-specific genomic, transcriptomic, or regulatory datasets—will be essential to improve model portability and prevent systematic errors arising from mismatched genome architectures.

For whitefly resistance breeding, gLLMs offer the potential to prioritize candidate genes, optimize gene-editing targets, and simulate resistance phenotypes under various genetic backgrounds see **Table 3**. When integrated with large multiomics and phenomics datasets, these models can support in silico screening of breeding populations, reducing reliance on extensive field trials. As gLMs are incorporated into breeding pipelines, explainable AI (XAI) tools will be essential. Breeders require interpretable outputs showing why a model selects certain variants, motifs, or pathways as important. Techniques such as attribution scoring, motif enrichment mapping, and regulatory grammar visualization can provide biological interpretability. Equally important is experimental validation: gLM-prioritized genes or variants must be confirmed through transcriptomic perturbation assays, allele-swap experiments, CRISPR knockouts, or virus accumulation assays to ensure that predicted effects reflect causal mechanisms rather than statistical artefacts. For practical deployment, gLM outputs must feed into a transparent decision pathway. Predicted regulatory variants, prioritized candidate genes, or ranked CRISPR targets can guide parent selection, marker development, or early elimination of low-value lines. Integrating computational predictions with targeted wet-lab assays and field validation allows breeders to triage thousands of potential edits or segregating alleles into a refined subset with demonstrable phenotypic impact. The successful deployment of gLLMs will depend on the availability of high-quality annotated genomes, interoperable data infrastructures, and breeder-friendly interfaces. Collaboration between computational biologists, breeders, and AI specialists will be essential to harness the full power of these models for predictive and prescriptive breeding in vegetable crops. Together, AI-driven platforms, multiomics insights, and genomic language models form a synergistic toolkit for next-generation breeding, enabling faster, smarter, and more resilient solutions to the persistent challenge of whitefly resistance. To further illustrate the landscape of AI-powered genomic tools, **Table 2** summarizes prominent Genomic Language Models (gLLMs) adapted for plant genomics and their relevance to whitefly resistance breeding. These models offer capabilities such as regulatory element prediction, variant prioritization, and simulation of gene network dynamics, supporting advanced breeding strategies for complex traits like insect resistance. A critical next step is rigorous benchmarking. To date, few studies have compared gLMs directly with established genomic prediction tools such as GBLUP, machine-learning regressors, CNNs, or standard Transformer models (Montesinos-López et al., 2025). Systematic evaluation across multiple environments, populations, and traits—including whitefly resistance components—is urgently needed to determine whether gLMs provide superior predictive accuracy or deeper biological insight. Finally, responsible use of gLMs requires careful consideration of data access, licensing, and global equity. Many high-performing genomic LLMs are proprietary, limiting their use by public-sector breeding programs, particularly in low- and middle-income countries. Differences in computational infrastructure, data availability, and licensing costs risk amplifying existing disparities. The development of open-source gLMs, transparent training datasets, and equitable data-sharing frameworks will be crucial to ensure that AI-enabled breeding benefits diverse vegetable systems worldwide.

**Table 3:** List of genomic large language models (gLLMs) for whitefly resistance breeding in vegetables.

Name of the LLM	Type	Key capabilities	Best for	Training data	Unique strengths	Limitations	Reference
AgroNT	Genomic	- Predicts regulatory elements- Estimates promoter strength- In silico mutagenesis- Zero-shot learning for orphan crops	- Crop improvement- Variant prioritization- Cross-species predictions	48 plant species (primarily crops)	- 2.5× faster SNP prioritization than GWAS- Adaptable to understudied crops	- High computational cost- Limited interpretability	Mendoza-Revilla et., 2024
PlantRNA-FM	RNA-focused	- Predicts RNA structural motifs- Identifies stress-response elements- Estimates translation efficiency	- RNA biology- Stress response studies- Crop adaptation research	54B RNA sequences from 1,124 plant species	- ChatGPT-inspired architecture for RNA- 89% accuracy in cross-species predictions	- Specific to RNA, may miss DNA-level insights	Yu et al., 2024b
ESM-2 (adapted)	Protein	- Predicts protein structure- Assesses mutation impacts- Infers protein-protein interactions	- Protein engineering- Enzyme optimization- Pathogen resistance	250M protein sequences (not plant-specific)	- Transferable to plant proteins- Enables 3D structure prediction	- Not plant-specific, may require fine-tuning	Lin et al., 2023
Geneformer (plant-adapted)	Single-cell	- Predicts gene network dynamics- Simulates genetic perturbations	- Cell-type specific studies- Developmental biology	30M single-cell transcriptomes (adaptation needed)	- Integrates spatial transcriptomics- Models cell-cell interactions	- Limited plant-specific training data	Avsec et al., 2021
DNABERT (plant version)	DNA-focused	- Identifies transcription factor binding sites- Predicts chromatin states	- Regulatory genomics- Epigenetic studies	Plant genome sequences (version-dependent)	- High accuracy in motif discovery- Adaptable to different plant species	- May struggle with very long-range interactions	Ji et al., 2021

## Challenges in breeding for whitefly resistance

Despite remarkable technological advances in breeding tools and molecular biology, the development of durable, commercially viable whitefly-resistant cultivars continues to face multiple scientific, logistical, and economic challenges. These constraints are particularly acute in vegetable crops like tomato, pepper, eggplant, and snapbean, where complex trait architecture, limited genetic resources, and evolving pest dynamics converge.

### Complexity of resistance traits

Whitefly resistance is typically polygenic, involving interactions among numerous loci that control traits such as trichome density, secondary metabolite production, and hormone signaling. Recent transcriptomic, metabolomic, and multiomics studies have begun to clarify this polygenic architecture by identifying coordinated defense signatures associated with resistance. Transcriptome profiling in tomato and pepper consistently shows upregulation of JA-responsive transcription factors, receptor-like kinases, and defense-associated secondary metabolism genes in resistant genotypes following *B. tabaci* feeding (Kong et al., 2025; Park and Ryu, 2014). Metabolomic analyses reveal characteristic shifts—such as elevated acylsugars, phenylpropanoids, flavonoids, and terpenoids—that correlate strongly with reductions in whitefly survival, fecundity, or feeding duration (Vosman et al., 2018; Kortbeek et al., 2021). When integrated with QTL mapping, these omics-derived signatures enable fine-scale prioritization of candidate genes within broad QTL intervals and reveal how multiple pathways—trichome development, hormone signaling, and specialized metabolism—act synergistically to produce quantitative resistance phenotypes. These systems-level insights are especially valuable for improving selection accuracy in breeding programs where individual loci have modest effects but collectively drive robust resistance. These traits are often influenced by genotype × environment (G×E) interactions, making them difficult to consistently phenotype and select under field conditions. In tomato, for instance, resistance based on acylsugar production requires multiple QTLs to be introgressed simultaneously, often with limited recombination due to linkage drag (Firdaus et al., 2013; Momotaz et al., 2010). Therefore, breeding strategies incorporating methods like recurrent selection are essential to effectively accumulate and fix these complex resistance traits.

### Trade-offs between resistance and agronomic performance

Introgressing resistance traits from wild relatives often come at the cost of reduced yield, poor fruit quality, delayed maturity, or loss of consumer-preferred traits. In pepper and eggplant, donor accessions with desirable resistance traits frequently show reduced vigor and low combining ability in hybrid backgrounds (Taher et al., 2020). Balancing resistance with productivity and marketability remains one of the central dilemmas in breeding programs. Field evaluations have repeatedly shown how these trade-offs manifest in commercial breeding lines. In tomato, for example, advanced lines carrying high-acylsugar QTLs from *S. pennellii* or *S. habrochaites* often exhibit sticky foliage, reduced fruit set under high temperatures, or yield penalties ranging from 8–20% compared with elite susceptible hybrids (Smeda et al., 2023). Similarly, pepper genotypes expressing strong whitefly antixenosis—frequently associated with elevated flavonoid or phenolic accumulation—have shown reduced fruit size or delayed maturity in multi-location trials (Sandra and Maharijaya, 2022). These observations highlight that metabolite-linked resistance, while effective against whiteflies, can impose allocation costs or alter source–sink dynamics. To overcome these challenges, strategies such as advanced backcrossing, marker-assisted background selection, genomic selection, and targeted gene editing are increasingly being employed to minimize linkage drag and accelerate the recovery of elite genetic backgrounds while retaining resistance loci (Samantara et al., 2022; MacNish et al., 2025).

### Biotype-specificity and durability of resistance

Whitefly populations are genetically diverse and rapidly evolving. Resistance that is effective against one biotype may break down when exposed to another, especially across regions or cropping seasons. The *B. tabaci* species complex includes over 40 cryptic species, including the highly invasive MED and MEAM1 biotypes, which differ in their virulence and virus transmission efficiency (Giorgini et al., 2023). As seen in tomato, resistance to TYLCV conferred by Ty-1 and Ty-3 loci varies depending on the infecting virus strain and whitefly biotype (Zamir et al., 1994). From an evolutionary ecology perspective, resistance durability is not solely a breeding challenge but also a vector population management problem. Continuous exposure of a dominant whitefly biotype to the same resistance sources exerts directional selection, favoring variants capable of overcoming plant defenses or transmitting virus more efficiently. This evolutionary pressure can accelerate resistance breakdown, especially in landscapes dominated by single resistant cultivars. Coordinated management strategies—such as rotating cultivars with distinct resistance mechanisms, integrating biological control agents, reducing volunteer hosts, and synchronizing cropping calendars—are essential to slow adaptive responses in whitefly populations. Long-term resistance stewardship therefore requires coupling genetic resistance with ecological and population-level monitoring frameworks. Durable resistance requires stacking of multiple, complementary resistance mechanisms and continual pest monitoring. In such scenarios, resistance strategies based on physical barriers—such as enhanced trichome density and cuticular traits—offer more stable protection across biotypes. Recurrent selection for polygenic traits like acylsugar production and trichome architecture, combined with genomic selection, could enable the accumulation of quantitative resistance alleles that are effective across a spectrum of whitefly variants.

### Environmental influence on resistance expression

Abiotic factors including temperature, humidity and light intensity significantly affect the expression of resistance traits like trichome development or metabolite biosynthesis. These factors may reduce resistance effectiveness under certain field conditions, leading to poor repeatability across environments. For example, trichome-based resistance in tomato and eggplant often declines under high temperatures or low light (Khan and Khan, 2018; Santegoets et al., 2021), and resistance in pepper was reduced in plants grown under salt stress (Caarls et al., 2024) necessitating multi-location and multi-season validation. Similarly, drought stress and altered photoperiods can reprogram hormone signaling pathways like jasmonic acid (JA) and salicylic acid (SA), which are integral to whitefly defense, thereby compromising resistance phenotypes. Moreover, genotype × environment (G×E) interactions may result in location-specific resistance breakdowns or inconsistent performance across growing seasons. This necessitates not only multi-location and multi-season field evaluations but also the integration of environmental response QTLs (eQTLs) in breeding pipelines. Recent advances in environmental modeling and high-throughput phenomics are enabling more climate-smart breeding decisions. Machine-learning models trained on multi-environment trial (MET) data combined with weather variables have been used to predict the performance of resistance-related traits—such as trichome density, chlorosis severity, or acylsugar accumulation—under projected temperature and humidity scenarios (Berlingeri et al., 2025). Deep learning applied to drone-based multispectral and thermal imaging can identify genotypes with stable physiological responses to combined abiotic–biotic stress, allowing breeders to detect alleles whose expression remains robust despite fluctuating field conditions (Pereira et al., 2025). Reaction-norm models and G×E-aware genomic selection pipelines can further rank genotypes by stability, helping identify whitefly resistance alleles that perform consistently across climate gradients. These tools collectively expand the breeder’s ability to forecast resistance durability and design climate-resilient ideotypes before large-scale field deployment. Advances in phenomics and environmental modeling can further support the selection of stable resistance alleles under dynamic field conditions. Long-term strategies should include screening under controlled abiotic stress simulations and stacking of biotic and abiotic resilience traits using genomic selection.

### Resource gaps in underutilized crops

Compared to tomato, crops like eggplant, pepper and snapbean are under-resourced in terms of genomic tools, phenotyping platforms, and curated germplasm collections (Gramazio et al., 2018). The lack of high-quality reference genomes, QTL mapping populations, and functional markers hampers the effective deployment of molecular breeding strategies. These gaps are often rooted in structural funding asymmetries and institutional priorities. Investment tends to favor globally traded, high-value commodities, while regionally important vegetables receive limited and fragmented support from national research systems. Many public breeding programs in low- and middle-income countries operate with short-term project funding, limited bioinformatics capacity, and restricted access to sequencing or phenotyping infrastructure, making it difficult to sustain long-term pre-breeding efforts or maintain diverse working collections. This institutional underinvestment perpetuates dependence on a narrow set of susceptible commercial varieties and slows the discovery and deployment of novel resistance sources in underutilized crops. Public-private partnerships and international consortia are needed to address these gaps, particularly for crops with regional importance but limited global investment. International data-sharing frameworks modeled after CGIAR-style platforms or open-source breeding consortia could play a pivotal role in closing these resource gaps. Shared repositories for reference genomes, pangenomes, QTL maps, and standardized phenotypic datasets for eggplant, pepper, and snapbean would reduce duplication of effort and lower the entry barrier for national programs. Open-access germplasm panels with accompanying genomic and phenotypic data, coupled with FAIR (Findable, Accessible, Interoperable, Reusable) data principles, would enable collaborative discovery of whitefly resistance loci and accelerate their deployment across regions. Coordinated networks that align funding agencies, public breeders, and private seed companies around common pre-breeding goals could ensure that tools, data, and training opportunities are equitably distributed rather than concentrated in a few well-resourced institutions.

In summary, overcoming these multifaceted challenges requires a coordinated effort combining robust phenotyping, high-resolution genotyping, genome editing, and AI-powered predictive tools. Embedding resistance breeding within an integrated pest management (IPM) framework will also be essential to enhance durability, reduce selection pressure on pests, and align with sustainable agriculture goals.

## Future prospects and considerations

The future of breeding for whitefly resistance in vegetables lies in the convergence of precision phenotyping, high-resolution genomics, and AI-driven predictive analytics. Addressing current limitations while harnessing emerging technologies will shape the next generation of resilient vegetable cultivars, particularly in tomato, pepper, eggplant, and snapbean.

### Integration of multi-trait breeding strategies

Moving beyond single-gene or monogenic resistance, future breeding efforts must integrate multiple resistance mechanisms—antixenosis, antibiosis, and tolerance—within elite backgrounds. Gene pyramiding, informed by marker-assisted and genomic selection, will be essential for conferring durable, broad-spectrum resistance (Dormatey et al., 2020; Merrick et al., 2021; Kumaraswamy et al., 2024). To complement this, recurrent selection can be employed to gradually accumulate favorable alleles across multiple loci, especially for polygenic traits such as whitefly resistance. This cyclical selection approach is particularly effective when phenotypic expression is influenced by genotype-by-environment interactions and when donor sources carry linkage drag or suboptimal horticultural traits. It is essential that this strategy also ensure compatibility with key agronomic traits such as yield, flavor, and shelf life. Given the inherent trade-offs between defense and productivity, future breeding pipelines must incorporate multi-objective optimization frameworks capable of balancing resistance and agronomic performance simultaneously. Selection index approaches—where whitefly resistance, yield, quality, and stress resilience are weighted according to breeding goals—offer a rigorous way to manage competing trait demands. Weighted genomic selection (wGS) further extends this by assigning differential weights to resistance- and yield-associated markers, allowing breeders to modulate selection pressure dynamically based on market needs, climate risks, or production constraints. Such frameworks ensure that gains in resistance do not come at the unacceptable cost of yield penalties or compromised fruit quality. Achieving reliable multi-trait improvement will require standardized phenotyping protocols across locations, seasons, and imaging platforms to ensure data compatibility for advanced computational models. Harmonized scoring scales for resistance traits (e.g., chlorosis indices, trichome metrics, virus severity ratings) and structured metadata standards are essential for enabling machine learning models to integrate multi-trait, multi-environment datasets. Emerging analytic tools—including multi-trait genomic prediction models, reaction-norm GS, and deep learning architectures that fuse phenomics, metabolomics, and environmental variables—can identify resistance alleles that provide optimal performance across diverse field conditions. Developing and adopting these integrative models will be crucial for climate-smart, multi-objective breeding strategies.

### Advanced genome editing and gene stacking

CRISPR/Cas systems offer unparalleled precision for editing resistance-related genes. Multiplexed editing, where multiple loci are modified simultaneously, opens avenues for combining virus resistance (e.g., Ty genes) with insect deterrence (e.g., acylsugar QTLs). Beyond gene stacking, genome editing provides the ability to decouple resistance traits from undesirable agronomic traits by precisely modifying or eliminating adjacent deleterious alleles without disrupting the resistance gene function. Emerging platforms such as base editing and prime editing further enhance this potential by enabling targeted nucleotide substitutions or insertions without inducing double-strand breaks, reducing off-target effects and allowing for more refined trait improvement. These tools will be critical in overcoming traditional trade-offs between resistance and traits such as fruit quality, yield, and shelf life, thereby improving both durability and marketability of whitefly-resistant cultivars (Ahmad, 2023). Despite these advantages, several practical challenges constrain the deployment of advanced genome-editing systems in vegetable crops. Regulatory ambiguity persists globally: some jurisdictions exempt certain gene-edited plants from GMO regulation, while others classify even single-nucleotide edits as transgenic, creating uncertainty for commercial release and international seed movement. Technical limitations also remain substantial in recalcitrant species such as pepper and eggplant, where low transformation efficiency, poor regeneration capacity, and strong genotype dependence limit routine editing. Rigorous off-target verification protocols—including whole-genome resequencing, amplicon-based deep sequencing, and computational off-target prediction—are increasingly required to demonstrate editing precision and build regulatory and consumer confidence. Computational tools, including genomic language models (gLMs) and AI-driven design platforms, can help overcome some of these constraints by improving guide RNA selection, predicting repair outcomes, and assessing off-target risks with higher accuracy than conventional rule-based scoring systems. These models can learn regulatory and sequence-context patterns that influence Cas nuclease activity, enabling the design of gRNAs that maximize editing efficiency while minimizing unintended edits. Incorporating such predictive tools into editing pipelines will be essential as multiplex and network-level editing become more common in whitefly resistance breeding.

Ultimately, the success of edited lines depends on field-level validation. Edited plants must be evaluated across locations and seasons to confirm that engineered resistance remains stable under variable abiotic and biotic pressures and does not introduce unintended yield penalties or fruit quality defects. Multi-environment testing is especially critical for traits such as acylsugar-mediated resistance or trichome modifications, which are sensitive to temperature, light, and nutrient regimes. Only through rigorous agronomic evaluation can genome-edited germplasm be advanced with confidence into commercial breeding pipelines.

### Harnessing AI and recurrent selection with genomic prediction

The growing availability of genotypic, phenotypic, and environmental datasets offers fertile ground for AI-powered tools to predict resistance phenotypes, optimize cross combinations, and simulate breeding outcomes. Genomic language models (gLLMs) and machine learning algorithms will support in silico design of sgRNAs, prioritize candidate genes, and guide breeding pipelines (Toufiq et al., 2023). To enable broad and equitable adoption of AI-driven breeding, there is a pressing need for shared data infrastructures that link genotypic, phenotypic, and environmental datasets across institutions and regions. Open-source AI pipelines—complete with standard metadata formats, transparent training datasets, and reproducible workflows—would democratize predictive breeding and allow public breeding programs with limited computational resources to benefit from advanced genomic prediction and decision-support tools. Such collaborative infrastructures mirror successful models in other domains (e.g., CGIAR’s integrated datasets) and could accelerate innovation in crops where data remains sparse.

While AI-enabled genomic prediction offers remarkable accuracy and speed in selecting superior genotypes, recurrent selection remains a valuable strategy for continuously accumulating favorable alleles, particularly for polygenic traits such as whitefly resistance. Recurrent selection allows the breeder to capture additive and epistatic effects across cycles, which can then be complemented by genomic prediction to increase selection efficiency. Integrating both approaches—using AI to predict and simulate performance, while implementing recurrent cycles of selection in real populations—can enhance long-term genetic gain and stability of resistance traits. This synergy is especially important in resource-constrained breeding programs where robust phenotypic evaluation and population improvement must proceed hand-in-hand. As AI becomes increasingly embedded in breeding pipelines, model interpretability is essential. Breeders must understand why an algorithm selects a specific cross, variant, or genotype as superior. Explainable AI (XAI) tools—such as feature importance scores, saliency maps, and attention-weight visualization—enable users to connect AI predictions with underlying biological mechanisms (e.g., trichome-regulating genes, metabolite pathways, or QTL intervals). Interpretability not only improves trust but also facilitates informed breeding decisions that prioritize biologically meaningful targets rather than black-box correlations. Equally important is the validation of AI models under real-world variability. Predictive models trained on controlled-environment or single-location datasets may fail when exposed to heterogeneous field conditions—including fluctuating whitefly pressure, mixed virus infections, and abiotic stresses. Establishing benchmarking protocols that compare AI predictions against multi-season, multi-location performance is critical for ensuring robustness. These benchmarks should incorporate environmental covariates, G×E-aware modeling, and independent validation populations to determine whether AI predictions translate into stable field performance.

### Exploiting wild relatives and landraces

Wild species like *S*. *galapagense and S. habrochaites* remain invaluable sources of whitefly resistance. Comprehensive characterization, domestication-focused pre-breeding, and targeted introgression will facilitate the translation of these traits into cultivars. In tomato, several wild species have been well studied for traits such as acylsugar production and trichome density; however, in crops like pepper and eggplant, wild relatives remain underexplored and poorly characterized for whitefly resistance. More systematic phenotyping, genotyping, and evaluation under whitefly pressure are needed to unlock their breeding potential. The application of speed breeding, combined with genomic tools, can significantly accelerate pre-breeding and introgression efforts in these crops. To fully exploit these reservoirs of resistance, establishing international pre-breeding consortia modeled after CGIAR and other global collaborative platforms will be essential. Such networks could coordinate systematic screening of wild relatives and landraces across diverse environments, share high-resolution phenotypic and genotypic datasets, and assemble species-wide pangenome databases for tomato, pepper, eggplant, and snapbean. These pangenomes would allow breeders to map structural variants, presence–absence variants, and novel alleles underlying whitefly resistance that are absent from current reference genomes, thereby accelerating the uptake of beneficial diversity into elite backgrounds. Incorporating evolutionary ecology principles can further enhance the identification of robust resistance traits. Wild relatives often harbor defenses shaped by persistent herbivore pressure, making them valuable models for detecting alleles that confer stability across fluctuating climates and whitefly biotypes. Metabolomic profiling of wild accessions—particularly of acylsugars, sesquiterpenes, flavonoids, and phenylpropanoids—can help identify biochemical signatures that are both heritable and resilient to environmental variation. These combined approaches allow breeders to distinguish transient, stress-induced defenses from genetically stable mechanisms suited for long-term crop improvement. Adaptive domestication frameworks that integrate speed breeding with iterative phenotypic and genomic selection can streamline the conversion of wild germplasm into agronomically viable pre-breeding material. Early cycles focus on retaining key resistance alleles, while subsequent rounds emphasize recovery of desirable horticultural traits such as plant architecture, yield components, and fruit quality. This staged domestication process reduces linkage drag, shortens breeding cycles, and enables the parallel improvement of resistance and agronomic performance.

### Crop-specific future breeding priorities for whitefly resistance

- **Tomato:** Advance fine-mapping and deployment of *Ty* and acylsugar-related QTLs; apply CRISPR-based editing to optimize trichome regulation and reduce linkage drag while maintaining fruit quality and yield.
- **Pepper:** Identify and validate QTLs associated with trichome density and secondary metabolite pathways; improve genotype-independent transformation systems to enable routine gene editing.
- **Eggplant:** Characterize resistance-related metabolites from *S. incanum* and *S. torvum*; integrate speed breeding and HTP for rapid screening under variable whitefly pressures.
- **Snapbean:** Expand screening for antibiosis and tolerance traits using genomic selection and AI-assisted phenotyping; develop biotype-specific resistance assays for durable deployment.
- **Cross-cutting:** Strengthen multiomics and methylome-guided approaches to identify regulatory hubs linked to inducible and climate-resilient resistance.

### Climate-resilient breeding frameworks

Emerging environmental fluctuations—including rising temperatures, erratic rainfall patterns, and prolonged drought are intensifying whitefly outbreaks and affecting crop physiology in ways that compromise existing resistance mechanisms. For instance, increased temperatures have been reported to reduce trichome density and disrupt the biosynthesis of secondary metabolites associated with whitefly resistance in tomato and eggplant, thereby lowering plant defense efficacy.

To ensure long-term durability of resistance, breeding programs must target genotypes that exhibit resilience to both pest pressure and abiotic stressors. This involves integrating resistance breeding with physiological traits related to heat and drought tolerance. Multi-environment trials and stress-adaptive phenotyping can help identify lines with stable resistance performance. Additionally, phenomic and transcriptomic data collected under controlled and field stress conditions can reveal co-regulated genes and pathways underpinning dual tolerance traits. The use of genomic selection tools trained on such datasets can enhance the identification of genotypes with high adaptive potential across environments.

Eco-physiological modelling, when combined with climate projection data, offers the ability to predict how rising temperatures, heat waves, and changing precipitation patterns will affect whitefly–crop interactions. Process-based models can simulate pest population dynamics, virus transmission rates, and plant defense responses under future climate scenarios, helping breeders prioritize genotypes with stable resistance across these changing conditions. Integrating these models into genomic selection pipelines allows for the identification of genotypes that will maintain resistance under predicted environmental stresses, effectively anticipating future challenges in whitefly management. Co-selection for both abiotic and biotic stress tolerance is essential for creating climate-resilient cultivars. Multi-environment, data-driven breeding models can integrate phenotypic data from diverse environmental conditions to identify genotypes that combine resistance to whiteflies with tolerance to drought, heat, and salinity. Genomic selection models that incorporate environmental covariates, such as temperature or soil moisture, will allow breeders to select for plants that can withstand multiple stressors simultaneously, increasing the likelihood of success across variable field conditions. In addition to genetic and physiological traits, epigenetic mechanisms such as DNA methylation, histone modification, and small RNA-mediated regulation play important roles in plant adaptive responses to fluctuating environments. Recent studies suggest that stress-induced epigenetic changes can enhance plant resilience by reprogramming defense pathways, enabling plants to respond more effectively to subsequent stresses. Incorporating epigenomic profiling into breeding programs could help identify epialleles that confer long-term stress tolerance, providing breeders with an additional layer of heritable variation for adaptive resilience under unpredictable climate conditions. Developing stress-resilient cultivars that maintain productivity and pest resistance under adverse growing conditions is essential to future-proof vegetable cropping systems in vulnerable production zones.

### Investment in understudied crops

Compared to tomato, crops like pepper, eggplant and snapbean remain under-resourced in terms of genetic and genomic tools. Future efforts should prioritize sequencing, QTL mapping, and trait validation in these crops. Coordinated international initiatives can support data generation, germplasm exchange, and platform development. The creation of shared genotyping platforms and regional breeding networks, coordinated through international organizations such as CGIAR, FAO, or allied public–private partnerships, would substantially accelerate progress in these understudied crops. Centralized or federated genotyping services, coupled with open-access databases, could reduce costs and ensure that breeders across regions can access high-quality genomic data. In parallel, capacity-building initiatives focused on phenomics, bioinformatics, and genome-editing technologies are urgently needed in developing regions to enable local breeding programs to fully utilize emerging tools. Training local scientists, strengthening institutional infrastructure, and promoting equitable data-sharing frameworks are prerequisites for sustainable adoption of advanced breeding technologies and for ensuring that innovations in whitefly resistance benefit diverse production systems rather than a limited set of well-resourced programs.

### Compatibility with biocontrol organisms

Natural enemies are an important component of integrated pest management. Similar to the fact that some cultivars and are less accepted by whiteflies than others, natural enemies of whiteflies may or may not be impacted in their attractance to and foraging among cultivars of certain vegetables (Van Lenteren et al., 1989; McAuslane et al., 2000; Jones et al., 2024). Whenever plants deter natural enemies, the contribution from the natural enemies are thereby decreased. Conversely, neutral or positive effect on the natural enemy is positive for genotypes that are resistant to whiteflies. Therefore, it may be of value to consider any impact on the natural enemies when breeding for resistant to whiteflies. To capture these dynamics, ecological field trials that explicitly evaluate plant–whitefly–natural enemy tri-trophic interactions are needed. Such trials move beyond single-pest assays and assess how resistance traits influence predator and parasitoid abundance, foraging efficiency, and pest suppression under realistic field conditions. Incorporating natural enemy performance metrics—such as parasitism rates, predator retention, and biological control efficacy—can reveal trade-offs or synergies that are not apparent in laboratory-based resistance screens. In this context, resistance breeding can benefit from the adoption of ecological selection criteria, where candidate genotypes are evaluated not only for reduced whitefly infestation but also for their compatibility with beneficial insects. Resistance mechanisms that repel whiteflies without disrupting natural enemy behavior, or that actively enhance biological control, should be prioritized over traits that provide resistance at the expense of ecosystem services. This shift aligns resistance breeding with integrated pest management and agroecological principles. Combining volatile organic compound (VOC) profiling with behavioral assays offers a promising approach to identify genotypes that support both resistance and biological control (Bleeker et al., 2009). Plant volatiles play a key role in mediating natural enemy attraction, and resistance-associated changes in secondary metabolism can alter these chemical signals. Integrating GC–MS–based volatile analysis with olfactometer or field-based behavioral assays can help pinpoint genotypes whose volatile blends deter whiteflies while remaining attractive to predators and parasitoids, thereby enabling the simultaneous optimization of host resistance and biocontrol efficacy.

### Translational research and farmer-centric approaches

Finally, it is essential that resistance breeding extend beyond the laboratory to ensure real-world impact. This includes participatory breeding, validation under diverse field conditions, and integration with pest management strategies. Prioritizing farmer-preferred traits will facilitate adoption. The future of whitefly resistance breeding in vegetable crops depends on the fusion of biological understanding with technological innovation. Interdisciplinary collaboration and a proactive, systems-level approach will be pivotal in delivering climate-smart, pest-resilient, and high-performing cultivars. It is paramount for researchers to continuing to recognize the value of genotypes with partial whitefly resistance as opposed to the desired complete resistance. For resistance breeding to translate into widespread adoption, economic feasibility and seed system readiness must be explicitly considered. Cost–benefit analyses that quantify reductions in pesticide use, yield stability under whitefly pressure, and input cost savings relative to seed price premiums can provide compelling evidence for adoption by farmers and policymakers. Strengthening local and regional seed systems—through support for seed multiplication, quality assurance, and distribution—is equally critical (Directive and EC, 2009). Policy incentives, such as support for integrated pest management adoption, subsidies for resistant seed, or recognition of reduced pesticide footprints, can further accelerate the uptake of whitefly-resistant cultivars. Farmer participatory trials play a central role in bridging breeding outcomes with on-farm realities. On-farm evaluations allow breeders to assess not only resistance efficacy but also labor requirements, compatibility with existing pest management practices, market acceptability, and overall profitability. Feedback from farmers on fruit quality, shelf life, and consumer preferences can guide selection decisions early in the breeding pipeline, reducing the risk of developing technically resistant but commercially unattractive cultivars. Ensuring equitable impact requires that resistance breeding be embedded within gender-responsive and smallholder-oriented frameworks. Women farmers often play key roles in vegetable production, seed selection, and marketing, yet their preferences and constraints are underrepresented in breeding objectives. Inclusive participatory approaches that consider gender-differentiated access to resources, labor dynamics, and decision-making authority can improve adoption and sustainability. Similarly, breeding targets must reflect the realities of smallholder systems, where affordability, risk reduction, and resilience often outweigh maximum yield potential.

## Concluding remarks

Whiteflies remain among the most persistent and damaging pests of vegetable crops such as tomato, pepper, eggplant, and snapbean. Despite more than six decades of research, progress toward durable and widely adoptable whitefly-resistant cultivars has been incremental. Recent advances in genomics, CRISPR/Cas-based genome editing, multiomics integration, and AI-driven predictive breeding provide powerful new opportunities, yet multiple biological, environmental, and practical constraints continue to limit their translation into the field.

Whitefly resistance is inherently complex and polygenic, strongly influenced by genotype × environment interactions and biotype variability within whitefly populations. Resistance introgressed from wild relatives frequently carries agronomic trade-offs, complicating the simultaneous improvement of resistance, yield, and market quality. Environmental stresses such as heat and drought further destabilize resistance expression, while under-resourced crops—including pepper, eggplant, and snapbean—lack the genomic tools, germplasm resources, and phenotyping capacity needed to fully exploit modern breeding technologies.

Future progress will require a pragmatic, systems-level approach that integrates conventional breeding with genome editing, recurrent selection, and genomic prediction, while explicitly managing trade-offs and environmental variability. Aligning resistance breeding with integrated pest management, stakeholder engagement, and robust seed systems will be essential. Ultimately, durable whitefly resistance will depend as much on ecological and socio-economic considerations as on technological innovation, requiring coordinated, farmer-centered strategies to achieve sustainable impact.
